# PLMFit: benchmarking transfer learning with protein language models for protein engineering

**DOI:** 10.1093/bib/bbaf381

**Published:** 2025-07-30

**Authors:** Thomas Bikias, Evangelos Stamkopoulos, Sai T Reddy

**Affiliations:** Department of Biosystems Science and Engineering, ETH Zurich, Basel, Switzerland; Botnar Institute of Immune Engineering, Basel, Switzerland; Department of Biosystems Science and Engineering, ETH Zurich, Basel, Switzerland; Botnar Institute of Immune Engineering, Basel, Switzerland; Department of Biosystems Science and Engineering, ETH Zurich, Basel, Switzerland; Botnar Institute of Immune Engineering, Basel, Switzerland

**Keywords:** protein language models, protein engineering, transfer learning, protein fitness, parameter efficient fine-tuning, benchmarking

## Abstract

Protein language models (PLMs) have emerged as a useful resource for protein engineering applications. Transfer learning (TL) leverages pre-trained parameters to extract features to train machine learning models or adjust the weights of PLMs for novel tasks via fine-tuning (FT) through back-propagation. TL methods have shown potential for enhancing protein predictions performance when paired with PLMs, however there is a notable lack of comparative analyses that benchmark TL methods applied to state-of-the-art PLMs, identify optimal strategies for transferring knowledge and determine the most suitable approach for specific tasks. Here, we report PLMFit, a benchmarking study that combines, three state-of-the-art PLMs (ESM2, ProGen2, ProteinBert), with three TL methods (feature extraction, low-rank adaptation, bottleneck adapters) for five protein engineering datasets. We conducted over >3150 *in silico* experiments, altering PLM sizes and layers, TL hyperparameters and different training procedures. Our experiments reveal three key findings: (i) utilizing a partial fraction of PLM for TL does not detrimentally impact performance, (ii) the choice between feature extraction (FE) and fine-tuning is primarily dictated by the amount and diversity of data, and (iii) FT is most effective when generalization is necessary and only limited data is available. We provide PLMFit as an open-source software package, serving as a valuable resource for the scientific community to facilitate the FE and FT of PLMs for various applications.

## Introduction

Protein language models (PLMs) are becoming a valuable tool in computational biology with applications such as protein structure and function prediction, design and engineering [1–3]. Leveraging transformer-based architectures [4] and inspired by large language models (e.g. ChatGPT [5], Llama [6], Falcon [7]), PLMs are trained on a large corpora of unlabeled protein sequences and are able to generate sequences of amino acids representing proteins with a high likelihood of folding, expression, and biological function. During this process, known as pre-training, multi-layered PLMs capture evolutionary [8] and structural [9] dependencies between amino acids by attempting to either reconstruct a corrupted sequence (i.e. masked language modeling) or predict the next residue (i.e. token) given the previous as context (i.e. autoregressive language modeling). Acquired knowledge is stored in the weights of the different PLM layers and can transform the input sequence into an information rich representation: protein sequence embeddings. As an alternative to encoding only amino acid sequence information (i.e. one-hot encoding, OHE) or including evolutionary information extracted from multiple sequence alignments (i.e. BLOSUM matrix) [10], PLM embeddings can be used as input features to train, typically shallow, machine learning (ML) models (i.e. artificial neural networks [ANNs] or convolutional neural networks [CNNs]) to solve a wide variety of protein characterization and engineering tasks [11–15].

Transfer learning (TL) leverages pre-trained parameters to train or adjust a different model to a novel task; TL can broadly be divided, based on the adaptation of the pre-weights or not, into two categories: feature extraction (FE) and fine-tuning (FT). In the context of PLMs, FE employs the retrieval of pre-trained weights from a PLM layer, which converts protein residues into evolutionary informed features [16] that can be used as inputs for training novel models. On the contrary, FT includes the joint optimization of PLM (or PLM fraction) weights with an untrained network (i.e. downstream head) using labeled data. To increase generative and downstream performance, PLMs continue to scale to a larger number of parameters and training sets, which poses technical challenges for implementing FT. Arbitrary retraining of PLMs, especially recently established large models (e.g. ESM-3 [17]), is computationally infeasible and can cause catastrophic forgetting of previously acquired knowledge [18]. Strategies adapted from natural language processing may be able to mitigate these issues. For example, parameter-efficient fine-tuning (PEFT) is able to adapt pre-trained models to a new domain with minimal adjustments to the original parameters. These techniques focus on optimizing only a small subset of newly added parameters, rather than retraining the entire network, allowing lower resource consumption and maintaining or improving performance on the specific task. Commonly used methods include low-rank adaptation (LoRA) [19], which involves adding low-rank trainable matrices in parallel with the transformer layers and adapter modules [20], which uses the injection of small neural network modules in between each layer of the pre-trained model.

Several studies propose pairing of TL techniques with PLMs to extract meaningful representation of proteins [3, 21, 22], this is accomplished mainly by using the embeddings extracted from the last layer of the PLM as input features to train novel ML models and by investigating the effect of PEFT for applications in protein engineering [23–25]. However, it is still unclear in which setups the exploitation of PLMs has a guaranteed benefit, as baseline models trained with simple encoding schemes (i.e. OHE) have been shown to overperform PLM-based methods in relevant protein engineering tasks [26]. Moreover, choosing the most appropriate TL methods is not straightforward. Multiple factors require calibration in order to optimally retrieve the stored information (e.g. extraction layer, downstream head architecture, FT hyperparameters and more). Additionally, TL is impacted by the amount, diversity, and quality of training data as well as access to hardware resources (i.e. memory and number of GPUs). Recent studies have evaluated the effectiveness of PEFT methods applied to PLMs for addressing biology-related tasks [27], or attempt to identify which specific layers might be most beneficial for embeddings extraction [22, 28]. However, analysis of PLM layer-specific performance including both FE and PEFT methodologies has yet to be reported. Additionally, comparative studies with baseline models and very large PLMs (> 5 billion parameters) are missing.

**Figure 1:**
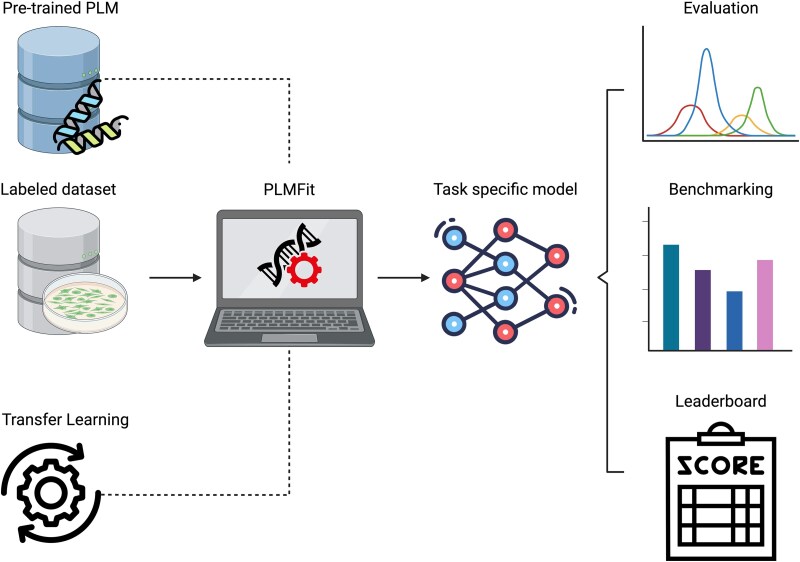
Overview of the PLMFit benchmarking analysis. PLMFit platform task-specific TL workflow. A pre-trained PLM can be paired with TL technique (FT or FE) with a labeled dataset. The resulting task-specific model is applied to a downstream task and benchmarked against alternative methods. PLM: Protein language models; TL: Transfer learning; FE: Feature extraction; LoRA: Low rank adaptation. Alt text – PLMFit overview.

We present PLMFit, a benchmarking framework that evaluates TL strategies using state-of-the-art PLMs for diverse protein engineering tasks. Applied to six public datasets covering property prediction and classification, PLMFit assesses over 3000 TL configurations by varying PLM architecture, layer selection, fine-tuning hyperparameters, and training procedures. Key findings include: (i) partial PLM usage can maintain performance, (ii) the choice between FE and FT depends on data volume and diversity, and (iii) FT excels in low-data, high-generalization scenarios. PLMFit offers practical guidance for leveraging PLMs based on task requirements and resources and is available as an open-access tool for applying TL to user data (Fig. 1).

## Results

### Utilizing datasets that represent a broad range of protein engineering tasks

To establish benchmarks on TL techniques, we used publicly available protein sequence-function datasets spanning various tasks and complexities, reflecting common protein engineering use cases. Each task includes different data splits, and we define a *configuration* as a specific PLM combined with TL methods and hyperparameters applied to a given training and evaluation setup. In protein engineering, training datasets typically fall into two main types: (i) protein sequence mutational landscape, which refers to the range of possible mutations that can occur, including substitutions, insertions, or deletions and the edit distance [ED] is the number of mutations a given variant has relative to a reference sequence (i.e. wild-type [WT]), where the number of mutations and distribution of residues remains relatively consistent and (ii) sequences of proteins from different families with a more diverse distribution of residues. For the first category, we utilized four fitness regression datasets parsed from the fitness landscape inference for proteins (FLIP) repository [15] and two protein–protein interaction (binding) classification datasets [29, 30] (Table 1). In this context, fitness refers to a protein’s ability to perform a function across different taxonomic groups and is influenced by multiple environmental factors, including stability, binding affinity, enrichment, foldability, and catalytic activity. For example, in antibody discovery, antibodies are experimentally mutated in various regions to investigate the change in binding affinity to a specific antigen [31]. For the second category (i.e. diverse datasets), we used the *Meltome* dataset from the FLIP repository, which includes melting temperature measurements for a diverse range of proteins and the secondary structure prediction (i.e. SS3) dataset [24], which involves predicting three-state secondary structures (helix, strand, coil) for sequences drawn from multiple protein families. Thus, protein sequences *in SS3* present considerable variation in residue composition and arrangement. We suggest that the complexity of each task is determined by both the quantity and diversity of the data available for training (i.e. training splits), as well as the nature of the testing data on which the prediction will be applied. Throughout the manuscript, we refer to a task using the dataset name and the split separated by a hyphen ‘-’ (e.g. *AAV-sampled*). Thus, two different tasks can refer to the same dataset but differentiate in complexity because of the training split (i.e *AAV-sampled* and *AAV-one versus rest).* We define tasks as simple, such as *AAV-sampled* and *GB1-three versus rest* due to the similarity in the distribution of the training and evaluation sets, which both consist of protein sequences with similar EDs from WT. In contrast, we define tasks as complex, such as *one versus rest* splits, as they involve limited training data that often consist of single point mutation variants (ED = 1) but require predictions on variants with a higher number of mutations (i.e. ED ≥ 2). We consider the *Meltome-mixed* and the *SS3-sampled* task to be complex due to the high variability in the sequences used. Additional details about the datasets and tasks used can be found in ‘Supplementary material’ section D (Datasets and downstream tasks).

**Table 1:** Summary of datasets and splits used for each dataset utilized in this study.

**Dataset name**	**Sequence length**	**Mutated region**	**Task type**	**Split**	**Training samples**	**Testing samples**
AAV	734–749	561–588	Regression (E_r_)	sampled	66 066	16,517
				one versus rest	1170	81,413
GB1	265	V39, D40, G41, V54	Regression (E_r_})	three versus rest	2968	5765
				one versus rest	29	8704
Meltome	20–750	-	Regression (T_o_)	mixed	24 817	3134
RBD	201	2–201	Binary classification (bind/escape)	one versus rest	875	217,484
Trastuzumab	449	99–108	Binary classification (bind/escape)	one versus rest	174	36,386
Secondary Structure	15–700	-	Token classification (helix/strand/coil)	sampled	10 556	357

### TL using a fraction of PLM impacts tasks’ performance

**Figure 2:**
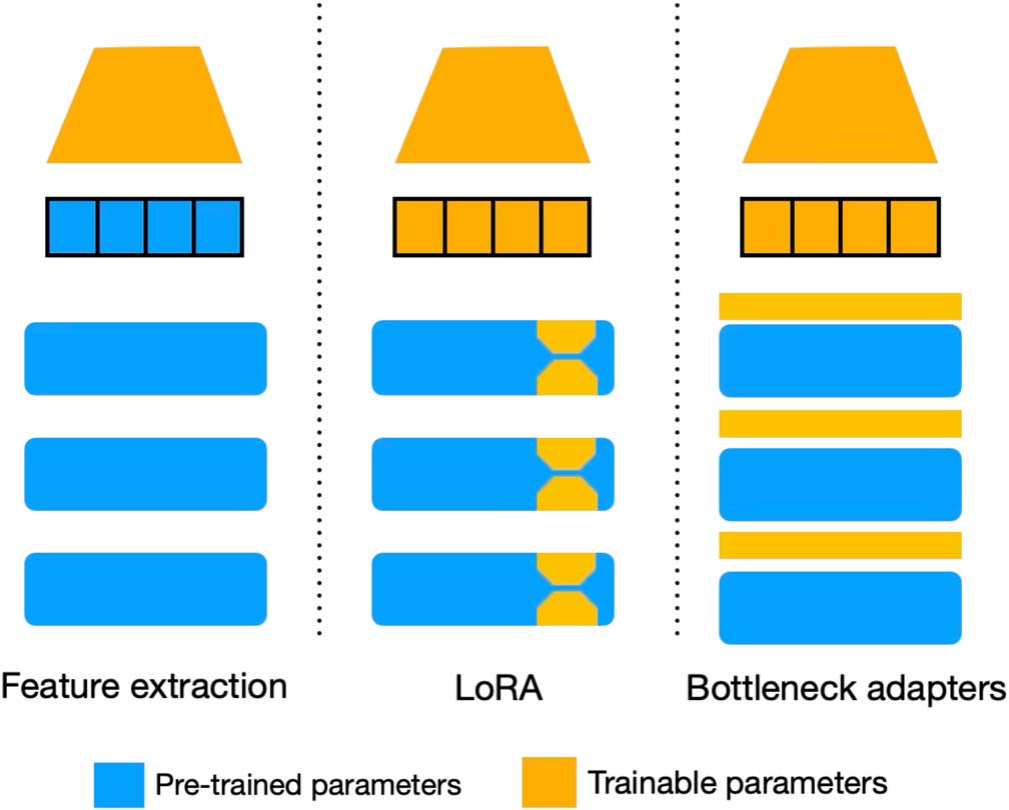
Transfer learning strategies used with protein language models. FE involves keeping the pre-trained model parameters frozen while using them to extract features. LoRA is parameter efficient FT technique that introduces trainable parameters into low-rank matrices within the layers to adapt the pre-trained model for a specific task. Similarly, bottleneck adapters inject trainable parameters within compact layers between the pre-trained parameters to adjust model outputs without full retraining. FE: Feature extraction; LoRA: Low rank adaptation. Alt text – PLM transfer learning strategies.

**Figure 3:**
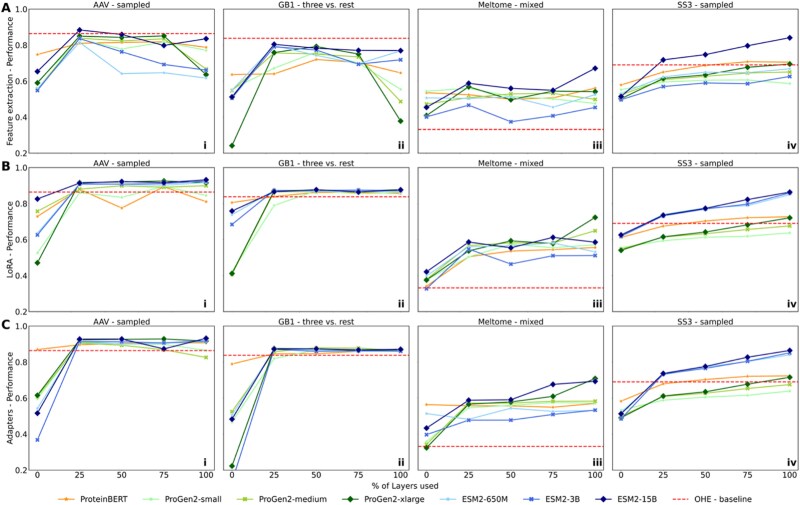
Performance analysis of TL models using different fractional layers of PLMs. TL is performed across three tasks, AAV-sampled, GB1-three versus rest, Meltome-mixed, and SS3-sampled (columns i-iii) using variable number of PLM layers paired with TL strategies, feature extraction, LoRA and adapters (rows A-C). PLMs are differentiated by color, and performance curves are displayed for each task. For each subplot, x-axis shows the percentage of layers used - 0 (corresponds to using only the first layer of the model), 25%, 50%, 75%, 100% (full model), and y-axis shows the Spearman’s correlation between the predicted and the ground truth values. The red dashed line represents a baseline model trained with the optimal hyperparameters using OHE of sequences, see methods. PLM: Protein language models; TL: Transfer learning; OHE: One hot encoding, FE: Feature extraction; LoRA: Low rank adaptation. Alt text – PLM layer analysis.

We evaluated three TL approaches: FE, LoRA and adapters (Fig. 2) for their performance across four different tasks (i.e. *AAV-sampled, GB1-three versus rest, Meltome-mixed, SS3-sampled*) and across different layers (first layer only, 25%, 50%, 75%, 100%) from three PLM families (ESM, ProGen and ProteinBERT) (Fig. 3) (see Datasets & Methods). Particularly across the tasks *AAV-sampled* and *GB1-three versus rest*, we observed that performance plateaued when 25% of layers were used for all three TL methods, after which there were minimal gains or even drops in performance. This suggests that pre-trained parameters stored in the first quarter of a foundational model may be more suitable when used for tasks that include protein sequences with similar distribution (i.e. variants of a WT) (Fig. 3Ai-ii-Ci-ii). Using these early parameters provides a simpler representation that focuses on general features, making them better suited for capturing subtle differences among closely related sequences. Thus, the final layers of a PLM are not always the best choice for TL. This pattern is consistent across all TL configurations that involve sequences within the same ED distribution between the training and the testing set. For tasks involving diverse protein sequences such as *Meltome-mixed* (Fig. 3A-Ciii) and *SS3-sampled* (Fig. 3A-Civ), incremental performance benefits are exhibited when deeper layers are targeted for TL, indicating that the task’s complexity and sequence variability benefits from richer and more comprehensive representations from the deeper layers of PLMs. This hints that, for highly diverse and complex tasks, leveraging the full depth of larger models can provide a distinct advantage, as they capture more nuanced features and dependencies across diverse protein families. Performance differences among the various PLMs, despite their differing architectures and sizes, are relatively marginal. While larger models like ProGen2-xlarge and ESM2-15B perform slightly better in most setups, shallower models such as ProteinBERT and ProGen2-small achieve comparable results even on complex tasks. This suggests that the model size and architecture do not always dictate performance when effective TL techniques are applied. The choice of TL method and the number of layers used appears to have a greater influence on performance, except in the case of the *SS3-sampled*, where larger ESM models (i.e. ESM2-3B and ESM2-15B) demonstrate a notable improvement in performance (Fig. 3B-Civ).

### Fine-tuning yields substantial performance gain in complex tasks

**Figure 4:**
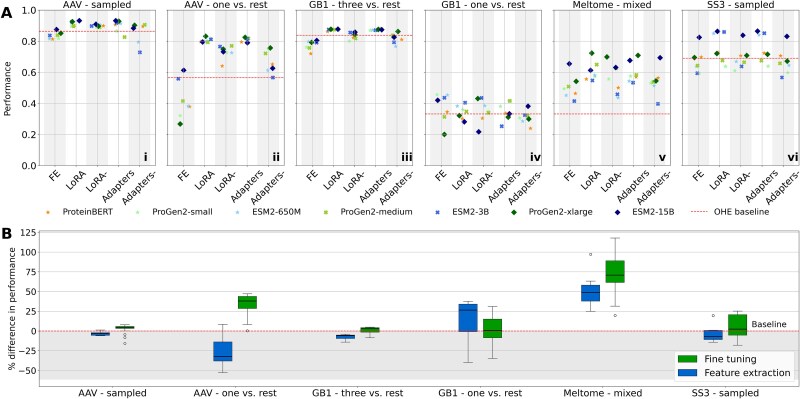
TL techniques performance comparison, in Spearman’s correlation (i-v), and macro accuracy (vi), across six different tasks. (A) Spearman’s correlation of the best performing PLM configuration with respect to the layer, downstream head and pooling method used for each TL technique (x-axis), is compared on: (i) *AAV-sampled*, (ii) *AAV-one versus rest*, (iii) *GB1-three versus rest*, (iv) *GB1-one versus rest*, (v) *Meltome-mixed* and (vi) *SS3-sampled*. Different PLMs are used: ProteinBERT, ProGen2 (small, medium, xlarge), ESM2 (650 M, 3B, 15B), with TL strategies including FE, LoRA, LoRA-, adapters, and adapters-. The red dashed line represents a baseline model trained with the optimal hyperparameters using OHE of sequences, see Methods. (B) Percentage difference in performance relative to baseline for FT and FE. Fine-tuning consistently yields larger performance improvements, particularly on more complex datasets like *Meltome - mixed*. Boxplots display variability in performance gains across tasks and TL methods. PLM: Protein language models; TL: Transfer learning; OHE: One hot encoding; FE: Feature extraction; FT: Fine-tuning; LoRA: Low rank adaptation. Alt text – Transfer learning performance analysis.

To assess TL effectiveness on six fitness prediction tasks (AAV-sampled, AAV-one versus rest, GB1-three versus rest, GB1-one versus rest, Meltome-mixed, SS3-sampled), we compared top-performing configurations—optimal layers and hyperparameters—across different PLMs (Table 2). Alongside standard LoRA and adapter methods, we evaluated lightweight variants (LoRA- and adapters-), which fine-tune only the final PLM layer (Table 3), unlike their full counterparts that modify multiple or all layers (‘Supplementary material’ section B, Figure S1). Overall, TL techniques outperformed baseline models trained with one-hot encoded (OHE) sequences (Fig. 4A). However, for AAV-sampled and GB1-three versus rest, performance gains were minimal, suggesting limited benefits of PLMs and TL in simpler tasks where training and test data share closely related sequence distributions (Table 1; Fig. 4A  i, 4A  iii). Conversely, TL methods demonstrated the highest performance gains for complex tasks consisting of training data with diverse protein sequences (*Meltome-mixed* and *SS3-sampled*), highlighting that PLMs excel in capturing distinct features across different protein families to accurately represent their amino acid sequences (Figs 4A  v.  3  vi). Similarly, TL methods also excelled in tasks where only single mutation variants were available during training, such as *AAV-one versus rest* and *GB1-one versus rest*, while sequences with higher ED were used for testing (Figs 4A  ii, 3A  iv). This suggests that TL on PLMs enhances model generalization by leveraging pre-existing knowledge encoded in these models, which can be particularly advantageous when dealing with limited or single mutation datasets. Interestingly, for ProteinBERT and ProGen2 family of PLMs, where FE did not surpass the baseline model (Fig. 4A  ii), all FT methods appeared to recover and enhance performance, thus underscoring the importance of co-optimizing pre-trained PLM weights while incorporating task-specific knowledge. This effect is particularly evident in the *AAV one versus rest* setup, where FE techniques averaged a Spearman’s correlation ($\rho$) of 0.44, which is 21.5% below the baseline ($\rho =$0.565), and whereas all FT models outperformed the baseline (average $\rho =$ 0.75, +34%).

Figure 4B illustrates the distribution of FE and FT effects on performance (percentage changes) compared to baseline models across each of the six tasks and highlights their impact on task performance in relation to the complexity of the task. For the *AAV-sampled* and *GB1-three versus rest* tasks, both FE and FT approaches perform similarly and close to the baseline with median performance difference of −3.2% / 3.95% and − 5.64% / 2.89% respectively (Table S1). However, as task complexity increases, such as in the *Meltome-mixed* task, FT yields substantial performance gain compared to FE, which can achieve an improvement of up to 117.82% over the baseline methods (Table S1). Simultaneously optimizing novel parameters with pre-trained weights, FT allows the models to extract deeper representations from the PLMs, enabling more effective learning from diverse and complex protein sequences. These results suggest that complex tasks benefit significantly from pre-training, which leverages the full capacity of the PLMs to handle intricate sequence variability and relationships.

PLMs did not present substantial differences in performance despite differences in protein families and data scale (Table 2). This suggests that the PLM architecture and pretraining carries less significance than the TL technique choice and calibration. However, in the *Meltome-mixed* and *SS3-sampled* task we observe that the larger models (ProGen2-xlarge, ESM2-3B, ESM2-15B) produce superior performance, which is anticipated, as an increased number of parameters enhances the model’s capacity to represent the diversity of proteins in this task. These observations underscore that high performance can be attained by leveraging smaller models, provided that TL is applied effectively. The TL configuration that performed best for each task is presented in Table 2. Analytical data are presented in ‘Supplementary Material’ section A (Extended results tables, Figure S2-S9).

**Table 2:** Scoreboard of the best transfer learning configuration results for each task.

**Task**	**Score**	**Metric**	**Best configuration**				
			**PLM**	**TL-method**	**Layers used**	**Pooling**	**Downstream head**
AAV one-vs-rest	0.8315	Spearman’s Corr.	ProGen2-xlarge	LoRA	quarter3	cls	linear
AAV sampled	0.932	Spearman’s Corr.	ESM2-15B	Adapters	last	mean	linear
GB1 one-vs-rest	0.4571	Spearman’s Corr.	ProGen2-small	FE	quarter3	mean	linear
GB1 three-vs-rest	0.8791	Spearman’s Corr.	ProGen2-medium	Adapters	middle	cls	linear
Meltome mixed	0.7232	Spearman’s Corr.	ProGen2-xlarge	LoRA	last	mean	linear
RBD one-vs-rest	0.5545	MCC	ProGen2-small	LoRA	middle	mean	linear
Trastuzumab one-vs-rest	0.3898	MCC	ProGen2-xlarge	LoRA-	quarter1	cls	linear
SS3 sampled	0.8644	Macro Accuracy	ESM2-15B	Adapters	last	-	linear

**Table 3:** Model configurations for Lora- and Adapters-.

**Tasks**	**PLM**	**Method**	**Performance**	**GPU RAM requirements (GB)**	**GPU Used**
AAV - sampled	ESM-3B	LoRA	0.9	28	NVIDIA A100 80GB (x3)
		LoRA-	0.9	6.4	NVIDIA Quadro RTX 6000 24G (x2)
		Adapters	0.9	29	NVIDIA A100 80GB (x4)
		Adapters-	0.6	6.5	NVIDIA Quadro RTX 6000 24G (x2)
GB1 - three versus rest	ESM2-15B	LoRA	0.9	48	NVIDIA A100 80GB (x4)
		LoRA-	0.9	6.5	NVIDIA Quadro RTX 6000 24G (x2)
		Adapters	0.9	52	NVIDIA A100 80GB (x4)
		Adapters-	0.8	7.2	NVIDIA Quadro RTX 6000 24G (x2)
Meltome - mixed	ProGen2-xlarge	LoRA	0.7	40	NVIDIA A100 80GB (x4)
		LoRA-	0.7	12	NVIDIA Quadro RTX 6000 24G (x2)
		Adapters	0.7	47	NVIDIA A100 80GB (x4)
		Adapters-	0.3	12.8	NVIDIA Quadro RTX 6000 24G (x4)

**Table 4:** Summary of protein language models used in this study.

**PLM**	**Type**	**No. of parameters**	**No. of layers**	**Embeddings Dim.**
ProteinBERT	MLM	92 M	12	768
ProGen2-small	CLM	151 M	12	1024
ProGen2-medium	CLM	764 M	27	1536
ProGen2-xlarge	CLM	6.4B	32	4096
ESM2-650 M	MLM	650 M	33	1280
ESM2-3B	MLM	3B	36	2560
ESM2-15B	MLM	15B	48	5120

### Fine-tuning performs better to higher mutation variants when only labels for single mutations are available

**Figure 5:**
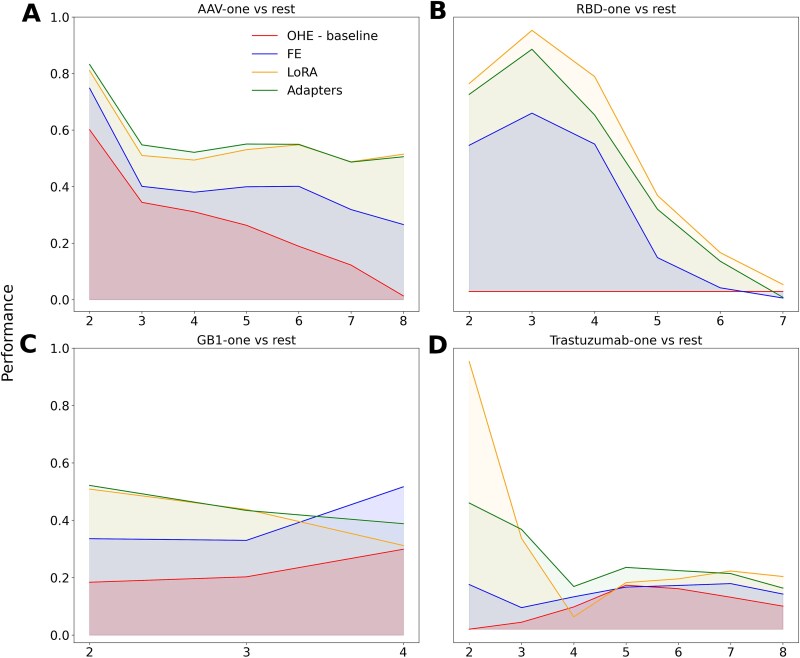
Performance of TL methods across different mutational edit distances for various datasets. (A-D) performance trends as a function of edit distance for four different datasets: (A) AAV-one versus rest, (B) RBD-one versus rest, (C) GB1-one versus rest, and (D) Trastuzumab-one versus rest. The y-axis shows model performance (Spearman’s correlation for GB1 and AAV dataset, MCC for RBD and Trastuzumab dataset), while the x-axis represents the ED from the reference sequence. Each line represents a different transfer learning strategy. PLM: Protein language models; TL: Transfer learning; OHE: One hot encoding; FE: Feature extraction; FT: Fine-tuning; LoRA: Low rank adaptation; ED: Edit distance; MCC: Matthews correlation coefficient. Alt text – Edit distance analysis.

Driven by the observation that fine-tuning PLMs can be particularly beneficial for *one versus rest* splits, we determined the performance of the best configuration from each TL technique (Table 2) across varying degrees of ED when trained only with single mutation variants. All TL-based models maintained more consistent performance levels as the ED increases compared to the respective baseline model, which rapidly loses efficacy (Fig. 5). Specifically for the AAV (Fig. 5A) and RBD (Fig. 5B) datasets, performance gradually declines at higher mutational levels, while the baseline models decline rapidly for the *AAV-one versus rest*. Model training was not achievable for any OHE baseline model for the *RBD-one versus rest* task (Fig. 5B), likely due to the high sparsity introduced by OHE and/or the lack of capacity in logistic regression and single-layer neural networks architectures. LoRA- and adapter-based models trained for the *GB1-one versus rest* and *Trastuzumab-one versus rest* tasks outperformed FE and the baseline across all ED, except at ED = 4 for the GB1 dataset (Fig. 5C, Fig. 5D); however, this deviation in performance can likely be attributed to the small training data size in the GB1 dataset (Table 1), raising concerns regarding the reliability of the observed trends. Similarly, for the Trastuzumab dataset, the limited number of sequences at lower ED (Table 1) compromises the reliability of predictions at higher ED, rendering the results less conclusive. These observations imply that leveraging single mutation labeled data to guide PLMs via TL can yield models that combine general knowledge acquired during pre-training (i.e. fitness and evolution) with task-specific data (e.g. experimental), making them capable of effectively generalizing to previously unseen protein sequences.

### Practical guidelines for applying PLMFit

Using the PLMfit platform, we have compiled practical guidelines for the research community to effectively apply TL techniques of FE and FT on PLMs. First, following the splitting of the dataset on training, validation, and testing sets, redundant (i.e. duplicates) and arbitrarily or mis-labeled (i.e. same amino acid sequence with different label) sequences must be removed to prevent ambiguity during training. Depending on the amount of diversity required for the task of interest, data can be further clustered by sequence identity with protein clustering tools (e.g. MMSeq2 [32] or kClust [33]). The main drivers of choosing a TL and PLM approach are the diversity of training data, the level of accuracy required for the task and amount of accessible resources. Generally, tasks performed on data with small sequence variation between the training and testing sets can benefit from training a custom model. However, choosing the optimal architecture and tuning the hyperparameters may not be straightforward, despite theoretically being able to bring higher results. In that case, FE using the 25% of a PLM with a linear downstream head could be sufficient and resource-efficient. Although fine-tuning a PLM can improve performance in these scenarios, the performance gains are often marginal.

For datasets characterized by single mutation variants (e.g. deep mutational scanning experiments), custom models typically struggle to generalize to variants with higher ED. In these instances, using LoRA to fine-tune a fraction (e.g. 25–75%) of a larger PLM (ESM2-15B, ProGen2-xlarge) is recommended to achieve better performance.

In more complex tasks involving diverse protein sequences (e.g. *Meltome-mixed*), model performance tends to scale with size and amount of trainable parameters. Consequently, employing TL on PLMs for this type of tasks can improve performance, as the extensive knowledge embedded in these models from large datasets of natural proteins can be effectively leveraged. Particularly, applying LoRA on large-scale PLMs, like ESM2-15B or ProGen2-xlarge, is likely to be the most effective method. However, when GPU availability or inference speed is a limiting factor, fine-tuning smaller PLMs, such as ESM-3B or ProGen2-medium, offers a practical alternative that can provide satisfactory performance.

## Materials and Methods

### Protein language models

TL techniques are applied to three state-of-the-art PLM families. Two BERT-based (ESM2 and ProteinBERT) and one GPT-based (ProGen2) foundational PLMs, pre-trained with MLM and CLM objectives respectively, are utilized. Different versions of these models are evaluated covering a broad range of architecture size with their layers size spanning from 12 to 48 layers, corresponding to 92 M up to 15B pretrained parameters respectively. Analytic overview of the PLMs assessed in this study are shown in Table 4.

### Fine-tuning and FT applied on varying fractions of PLMs

To assess the impact of TL on protein engineering tasks, we applied both FE and FT across multiple depths of pre-trained PLMs. Specifically, we extracted embeddings from five key positions within each PLM, the first layer, last layer, and intermediate layers corresponding to 25%, 50%, and 75% of the model’s depth, and used these as inputs for downstream tasks. In parallel, we fine-tuned the same models using parameter-efficient strategies by integrating either adapter modules or Low-Rank Adaptation (LoRA) at the same relative depths. Technical details of the TL methods, including architectural configurations, hardware requirements (Table S2–S9) and training procedures, are provided in the ‘Supplementary Materials’ section *C (Transfer Learning methods) and section G (Hardware resources)*

### Evaluation metrics and baselines

In this study, the performance of each TL-based model was compared to hyperparameter-tuned (Table S10, Table S11) logistic regression and multilayer perceptron (MLP) neural networks with one hidden layer. These comparisons were conducted using one-hot encodings (OHE). OHE is a method where each amino acid in a protein sequence is represented by a binary vector of length equal to the number of possible amino acids, where all elements are set to zero, except for the element in the position corresponding to the amino-acid in the protein sequence which is set to one. No information about the biochemical properties or evolutionary relationships between amino acids is captured using this encoding method. Following the encoding of each position with the amino acid corresponding one-hot vector, the input sequence represented by a 2-d matrix Z$\left( Z\epsilon{R}^{sequence\ length\ x\ embedding\ dimension}\right)$ is flattened before being used as input features to train the baseline models.

The evaluation metrics implemented in this study vary based on the nature of the task. For models trained on regression tasks, we utilized Spearman’s correlation coefficient ($\rho )$ to assess the strength and direction of the monotonic relationship between predicted and actual values (1).


$$ \rho =1-\frac{6\ {\sum}_1^n{\left({X}_i-\hat{X_i}\right)}^2}{n\ \left({n}^2-1\right)} $$

Equation (1). Spearman’s rank correlation coefficient ($\rho$) where $\rho \epsilon \left[-1,1\right]$, ${X}_i$ is the rank of the i-th element of real values, is the rank of the i-th element of predicted values, n is the total number of elements.

For binary classification tasks, we employed Matthew’s correlation coefficient (MCC), which provides a balanced measure of the quality of binary classifications, considering true and false positives and negatives (2)


$$ MCC=\frac{TP\cdotp TN- FP\cdotp FN}{\sqrt{\left( TP+ FP\right)\left( TP+ FN\right)\left( TN+ FP\right)\left( TN+ FN\right)}} $$

Equation (2). Matthew’s correlation coefficient (MCC) where TP, TN, FP, FN are the number of true positive, true negative, false positive and false negative prediction predictions respectively.

For the secondary structure prediction task, where classification predictions are made per token (i.e. for each residue in the protein sequence), we evaluated the model using the macro accuracy of all tokens in a sequence, which provides an averaged measure of the model’s accuracy across all residues irrespective of class imbalance (3).


$$ Macro\ accuracy=\frac{1}{N}{\sum}_{i=1}^N\frac{1}{L_i}{\sum}_{j=1}^{L_i}I\left({y}_{ij}=\hat{y_{ij}}\right) $$

Equation (3). Macro accuracy, where $N$ is the total number of protein sequences in the dataset, ${L}_i$ is the length of the i-th protein sequence, ${y}_{ij}$ and $\hat{y_{ij}\ }$are the true and predicted labels for the j-th residue in the i-th sequence, respectively, and $I$($\cdotp$) is an indicator function that equals 1 if the true label matches the predicted label and 0 otherwise.

## Discussion

In this study, we evaluated three TL approaches across six datasets, utilizing three families of state-of-the-art pre-trained PLMs. Each TL-based model was trained with varying parameters, including the number of layers, pooling methods, downstream architectures, and training hyperparameters. Our analysis extends prior work by exploring finer hyperparameter tuning and incorporating larger models. We compared performance against task-specific baselines trained with one-hot encoding (OHE) of sequences (Fig. 4). The results show that, when applied correctly, TL significantly benefits protein engineering tasks.

As PLMs are often used to represent sequences in evolutionary contexts or guide evolutionary processes [34], this work provides practical guidelines for leveraging stored knowledge. FE can incorporate evolutionary information into encodings, while full FT, though consistently improving performance, may not always justify its computational cost. Smaller models with optimal hyperparameters can match larger ones, making them advantageous given the growing model complexity. However, FT excels in few-shot and challenging tasks, allowing models to generalize by extracting deeper representations from PLMs. Performance gains from TL vary with task type and sequence diversity. Fine-tuning PLMs typically yields the most benefit when tasks involve sequences from diverse protein families, especially for properties conserved across proteins (e.g. folding, thermostability, or secondary structure) as observed in Meltome-mixed and SS3-sampled. In these cases, PLMs capture general biophysical principles like residue interactions or structural motifs. Conversely, tasks within a single protein family (e.g. AAV, GB1, or Trastuzumab) depend more on modeling fine-grained mutational effects linked to specific functions like binding or catalytic activity. Here, performance gains stem from adapting to localized sequence constraints.

A common technique in protein engineering is deep mutational scanning (DMS) [35] , which introduces single mutations and evaluates variants through specific assays. DMS is valued for its high-throughput nature and low experimental burden. Incorporating DMS data to train or inform PLMs with limited data can improve predictions on combinatorial libraries (edit distance >1)**.** Conversely, when ample data is available and test variants resemble training data, simpler models trained ab-initio may outperform PLMs with better efficiency. Thus, the best use of PLMs depends on data diversity, task nature, and available computational resources. Consistent with earlier studies, our results show the last PLM layer often underperforms.

While our study offers deep insights, more robust evaluation methods like k-fold cross-validation could enhance assessment. We also limited the exploration of LoRA ranks and complex adapter architectures due to computational costs. Future work should expand hyperparameter search to assess their full impact. This study used only sequence data, though the field is moving toward multimodal PLMs incorporating structural features [36, 37]. Dataset and task biases may affect results; broader and more diverse protein data are needed to validate findings. Ultimately, TL-based models must transition from computational validation to lab experiments for real-world impact.

We invite the community to contribute datasets, tasks, TL techniques, and PLMs to help establish benchmarks. We will continue updating Table 2 with the top TL–PLM combinations for each task.

We anticipate PLMFit will serve as a practical resource for researchers applying PLMs to protein engineering. Whether extracting embeddings or fine-tuning, PLMFit can streamline workflows and guide optimal parameter selection.

**Key Points**

- **Transfer learning with PLMs can be efficient by using only a fraction of model layers.** Our study reveals that using only a subset (as little as 25%) of a protein language model’s (PLM) layers can achieve comparable performance to full-model utilization, especially for tasks involving homologous protein sequences. Notably, ours is the only study to benchmark transfer learning on very large PLMs, including models with up to 15 billion parameters such as ESM2-15B. By fine-tuning only a fraction of layers, we demonstrate that it is possible to leverage the representational power of such large models while significantly alleviating the computational burden. This substantially reduces the number of trainable parameters, cuts down memory requirements and training time, and makes the use of PLMs feasible for smaller labs and individual researchers with limited computational resources.
- **The amount and diversity of training data dictate the optimal transfer learning strategy.** We provide a practical recipe for choosing between feature extraction (FE) and fine-tuning (FT), based on task complexity and data characteristics. For simple tasks with low sequence diversity, FE offers a computationally efficient solution with minimal loss in performance, while FT becomes essential for diverse datasets that require generalization across different protein families. This distinction is critical, as fine-tuning can be computationally expensive and time-consuming, whereas FE requires only a single forward pass to generate embeddings that can be reused across multiple downstream models.
- **Fine-tuning PLMs boosts generalization to protein variants with higher edit distances.** When fine-tuned using only deep mutational scanning (DMS) data containing single mutations, PLMs demonstrate the ability to generalize to combinatorial variants with multiple simultaneous mutations. This capability is enabled by their internal understanding of protein sequence patterns, allowing accurate predictions even when the model is trained on limited and low-edit-distance data. This can be particularly important as it helps reduce the experimental burden by minimizing the need to generate large combinatorial libraries for training.
- **Applying transfer learning only to the final PLM layer offers a strong performance-efficiency tradeoff.** Fine-tuning only the final layer of a PLM using memory-efficient strategies like LoRA—or adapters—delivers near-optimal performance while reducing GPU memory requirements by more than 50% (Table 3). This approach lowers the barrier for fine-tuning large PLMs, making transfer learning feasible on standard lab-grade hardware and promoting broader adoption.
- **PLMFit enables large-scale application of fine-tuning on PLMs with a reproducible software package.** Implementing fine-tuning workflows on PLMs is non-trivial due to model complexity, memory constraints, and the need for hyperparameter optimization. To address this, we developed PLMFit, an open-source benchmarking platform that abstracts these complexities and enables automated, reproducible fine-tuning experiments. Using PLMFit, we conducted over 3000 training runs across multiple models, TL techniques, and protein engineering tasks, providing a scalable and user-friendly tool for the broader research community.
- **PLMFit enables effective transfer learning for novel protein families by leveraging evolutionary and structural constraints.** PLMFit is a transfer learning framework that allows PLMs to be applied to a wide range of biological tasks. By capturing evolutionary and structural constraints from natural proteins, PLMFit is particularly well-suited for tasks involving novel protein families, where these global patterns help models generalize even with limited data.

## Supplementary Material

PLMFIT_Supplementary_Material_FINAL_SUBMISSION_bbaf381

## Data and materials availability

All codes and datasets are available at https://github.com/LSSI-ETH/plmfit. All datasets can also be found in their original repo: AAV/GB1/Meltome (https://github.com/J-SNACKKB/FLIP), Trastuzumab (https://github.com/dahjan/DMS_opt), RBD (https://github.com/LSSI-ETH/Omicron_DML), and SS3 (https://github.com/fengtuan/LIFT_SS).
