# Supplementary material for: PLMFit: benchmarking transfer learning with protein language models for protein engineering
Source: Brief Bioinform. 2025 Jul 30;26(4):bbaf381. doi: 10.1093/bib/bbaf381 (PMC12309243; doi:10.1093/bib/bbaf381)
Supplement: PLMFIT_Supplementary_Material_FINAL_SUBMISSION_bbaf381 [file plmfit_supplementary_material_final_submission_bbaf381.docx]

**Supplementary Material**

PLMFit : Benchmarking Transfer Learning with Protein Language Models for Protein Engineering

Thomas Bikias^1^, Evangelos Stamkopoulos^1^ and Sai. T. Reddy^1, 2^*

^1^Department of Biosystems Science and Engineering, ETH Zurich, Basel, Switzerland.

^2^Botnar Institute of Immune Engineering, Basel, Switzerland

*Corresponding author. Email: [sai.reddy@ethz.ch](mailto:sai.reddy@ethz.ch)

1. **Datasets and downstream tasks**

Fitness prediction data acquired from the widely adopted Fitness Landscape Inference for Proteins (FLIP[^15^](https://paperpile.com/c/UzxkQ5/Nfeqj)) repository which includes curated datasets with experimental measurements that map protein sequences with binding affinity and thermostability measurements for different protein families. Particularly, it consists of two datasets of variants starting from a wild type sequence, Adeno-associated virus capsid (i.e. AAV) and GB1 domain of immunoglobulin-binding protein G (i.e. GB1), which fitness corresponds to variant enrichment ratio[^39^](https://paperpile.com/c/UzxkQ5/F4dd)^,^[^40^](https://paperpile.com/c/UzxkQ5/RIZH) (i.e. E_r_ ). To simulate different training scenarios and use cases, each set is splitted in different setups. From the AAV dataset, we used “sampled” split, where sequences randomly chosen for training and testing, and “one vs rest” split, where single mutation variants used for training and the remaining sequences (up to 39 mutations) assigned to the evaluation set. From the GB1 dataset, “one vs rest” and “three vs rest” splits utilized for training the models with single and up to three edit distance variants respectively and the rest for testing (up to 4 mutations). For the thermostability prediction task, a dataset (i.e. Meltome) consisting of sequences clustered for 50% sequence similarity using MMSeq2[^32^](https://paperpile.com/c/UzxkQ5/xhIs9) have been assessed for the maximum temperature (i.e. T_o_) that they can perform their function (i.e. thermostability). A single split referred as “mixed” is used for this task, in which sequences randomly selected from 13 species are exploited during training.

The binding classification datasets used in this study consist of two distinct libraries[^29,30^](https://paperpile.com/c/UzxkQ5/9iEfb+SVmIX) screened for binding and escape against the human ACE2 receptor using yeast display and the HER2 antigen using mammalian display with hybridoma cells, respectively. The first library includes the mutational landscape of SARS-CoV-2 Omicron receptor-binding domain (RBD) variants, focusing on their binding and escape interactions with human ACE2. This library spans the entire 201 amino-acid RBD sequences, with mutations up to an edit distance of 7. The second library explores mutations within the complementarity-determining region H3 (CDRH3) of Trastuzumab, a therapeutic antibody, specifically assessing its binding affinity to the HER2 antigen. This dataset contains variants with up to 10 mutations in the Complementarity-determining regions (CDRH3) region.. We employ a similar splitting strategy as the FLIP repository for these datasets to define the binary classification tasks. For each, we define two splits: a "sampled" split, which consists of a random division of (70 % training, 15% validation, 15% testing), ensuring class balance in training and validation splits, and a "one-vs-rest" split, where only single-mutant variants (ED = 1) are used for training and validation, while variants with higher edit distances from the wild type (ED >1) are reserved for testing. Summary for all the data used in this study are shown in Table 1.

Finally, our analysis includes a secondary structure prediction (SS3) dataset for three-state secondary structure prediction (helix, strand, coil), derived from the nine-class DSSP 4.0[^41^](https://paperpile.com/c/UzxkQ5/tDrS) annotations: α-helix (H), β-bridge (B), strand (E), 310-helix (G), π-helix (I), turn (T), bend (S), loop (L) and poly-proline helix (P). Following the procedure described by Yang et al.[^24^](https://paperpile.com/c/UzxkQ5/t7qY6), these nine labels are mapped to three states: H, G, I, P as helix, B, E as strand, and T, S, L as coil. This simpler three-state representation is common for secondary structure classification tasks, as it captures broad structural categories while reducing labeling noise. The final “sampled” split, also adopted from the source, comprises 9504 sequences for training, 1052 for validation, and 357 for testing, making SS3 a challenging task due to sequence diversity and the comprehensive coverage of different structural states.

1. **Transfer Learning methods**

Transfer learning entails reusing or adapting a pre-trained model as the foundation for addressing a different, novel task. By leveraging the knowledge from the original domain, performance on the new task can be improved. Depending on whether pre-trained weights (fully or partially) are co-optimized with the parameters of the newly added task specific downstream head, TL can be divided to feature Extraction and fine-tuning. To adequately examine if TL-based computational approaches can benefit protein engineering, we investigated both as part of this study.

Feature extraction

Feature extraction, as a TL method, involves the transformation of the input sequence into a numerical representation after performing inference on a pre-trained PLM without altering its weights. Conceptually, obtained representations encapsulate information about the structure and evolution of the processed protein sequence and can be used as input features to train shallow models in downstream tasks. In this study, we employ a layer pruning analysis assessing multiple fractions of the foundational models by extracting embeddings from the first, the last and three intermediate layers corresponding to 25%, 50%, and 75% of the models’ size.

Fine-tuning

On the contrary, during fine-tuning, weights of a pre-trained model are adjusted to the specific downstream task. Weight adaptation can occur either entirely or selectively by retraining a fraction of the model’s parameters while retaining the remaining frozen during back propagation. The latter approach significantly reduces the number of gradients to be calculated, thereby decreasing the time and resources required for the fine-tuning process. Parameter-Efficient Fine-Tuning (PEFT) extends this concept by combining novel trainable networks with the original parameters. By using the pre-trained model only for inference and updating solely the weights of the smaller, newly added, modules, knowledge acquired during pre-trained can be leveraged to optimize the model for the specific task of interest. Motivated by state-of-the-art techniques in Natural Language Processing and methods that have been studied in the biology realm, this study proposes two PLM Fine-tuning approaches to establish benchmarks, bottleneck adapters and Low-Rank Adaptation (LoRA). Adapters are small architectures injected between the layers of a pre-trained PLM, allowing for efficient fine-tuning by freezing the original model's weights *W_o_* and only training the adapter parameters *W*$'$ (1a). For this study, adapters’ architecture proposed by Yang et al.[^24^](https://paperpile.com/c/UzxkQ5/t7qY6) is employed. Low-Rank Adaptation (LoRA) decomposes the weight matrix *W_o_* of a pre-trained model into two low-rank (*r* $\ll$ *d_k_* ) matrices *A* and *B*, significantly reducing the number of parameters to be trained (1b). LoRA modules applied on the pre-trained *W_q,k,v_* matrices of the attention heads in the different layers of the PLMs. Similarly, to feature extraction, the effect of adding FT-modules in different depths (first, last, intermediate; 25%, 50%, 75%) of PLMs is investigated. Additionally, to further decrease the amount of trainable parameters,we propose the addition of the respective modules only in the last layer of the foundation PLMs, namely LoRA- and the effect of this approach is being assessed as a trade-off between performance and computational efficiency.

1. $h_{i} = W'(W_{o}h_{i-1})$
2. $h_{i} = (W_{o}+AB)h_{i-1}$

Equation (S1). (a) Adapters fine-tuning where $h_{i}$ is the output of the current layer, $h_{i-1}$ is the input from the previous layer, $W_{o}$ is the weight matrix of the pre-trained model and $W'$ is a small trainable matrix, referred to as the adapter, introduced to fine-tune the transformation for task-specific requirements. (b) Low-Rank Adaptation (LoRA) where $h_{i}$ is the output of the current layer, $h_{i-1}$ is the input from the previous layer, $W_{o}$ is the weight matrix of the pre-trained model, $A$ and $B$ are the trainable low-rank matrices which product $AB$modifies $W_{o}$ for fine-tuning with minimal computational overhead.

1. **Applying LoRA only on the last layer provides a reliable tradeoff between memory constraints and model performance**

Our analysis demonstrated that FT techniques can yield superior results when adapting pre-trained PLMs for protein engineering tasks. Despite adopting PEFT methods, even when fine-tuning a small fraction of the model, the number of trainable parameters can still be large. This is due to the sheer size of models such as ESM2-15B and ProGen2-xlarge, which consist of 98 and 6.4 billion parameters, respectively. In such models, updating even a very small subset requires significant computational resources. Motivated by these challenges, we investigated the performance of FT modules added only to the final layer of the entire PLM, LoRA- and adapters- . Adopting this strategy, we reduced the number of trainable parameters by a fraction of the total layer number and reduced the computational and memory overhead. Comparative analysis reveals the performance (upper section) and memory usage (lower section) between LoRA, LoRA-, adapters, and adapters- across three tasks: *AAV-sampled*, *GB1-three vs. rest*, and *Meltome-mixe*d (Figure 6). While, both LoRA and adapters outperform their reduced counterparts, LoRA- and adapters-, the performance drops for *AAV-sampled* and *GB1-three vs rest* are marginal. Only *Meltome-mixed* exhibits a more notable decline when the adapters- method is applied. Importantly, both LoRA- and adapters- have significantly lower memory requirements, without a drastic performance loss. GPU RAM memory ranges from 6.5-12.8 gigabytes, compared to the standard application of these methods which require 28-52 gigabytes (Table 3). By reducing the memory requirements without significantly sacrificing performance, LoRA- and adapters- provide accessibility to larger PLMs and enable their FT without the need for expensive cloud computing services or specialized infrastructure. PLMs, training parameters and hardware resources used for LoRA- and adapters- for the comparative analysis and for the entirety of setups are shown in Table 3.

**
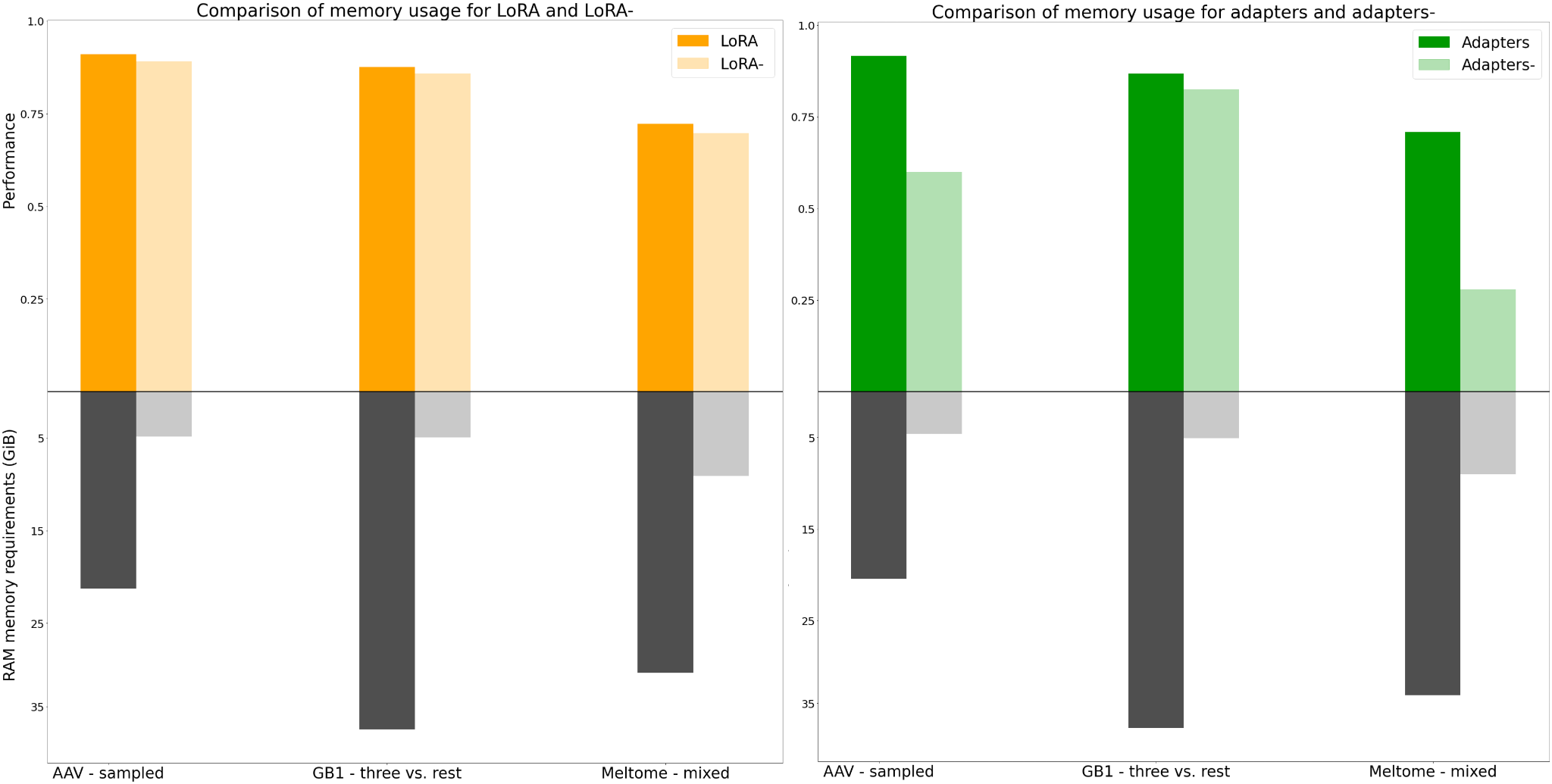
**

**Figure S1. Performance and memory trade-off between TL techniques utilizing full or last layer only PLMs.**The orange bars (dark for LoRA and light for LoRA-) and the green bars (dark for adapters and light for adapters-) show the performance (Spearman’s correlation) of the models on three datasets: *AAV-sampled, GB1-three vs. rest*, and *Meltome-mixed*. The y-axis represents performance on a scale from 0 to 1, where LoRA consistently outperforms LoRA- across all datasets. The gray part of the bars ( dark for default and light for - version) represents the memory requirements in gigabytes (GB) for each approach. LoRA requires significantly more memory compared to LoRA-, illustrating a trade-off between performance and memory efficiency.

LoRA: Low Rank Adaptation

1. **Training procedure**

Each architecture evaluated in this study, whether based on transfer learning (TL) or initialized with random weights, is trained until convergence, with early stopping based on validation loss as the stopping criterion. For each FE-based setup, we trained the downstream modules using embeddings generated from PLMs (ESM, ProGen, and ProteinBERT). To convert local embeddings into a global representation, we explored a parameter space that included two pooling methods: averaging the embeddings or utilizing the CLS token. For the downstream classifier, we evaluated two potential architectures—a logistic regression model and a one-layer artificial neural network (ANN)—to determine the optimal approach. All combinations of these settings were subject to hyperparameter tuning to ensure optimal training performance. For FT, we limited the downstream architecture to logistic regression due to the computational burden associated with running these experiments. The hyperparameter tuning space for FT was more constrained, as the training process is time- and resources- intensive; the parameter space was therefore defined based on configurations from existing literature[^24,42^](https://paperpile.com/c/UzxkQ5/NObat+t7qY6). Detailed descriptions of the training procedures and hyperparameter tuning can be found in the supplementary materials (Table S2).

Deepspeed package with CPU offloading and mixed precision training is utilized in PLMFit to manage computational resources[^34^](https://paperpile.com/c/UzxkQ5/qQiQ). Stage 3 of Deepspeed is applied, with smaller reduce and all-gather bucket sizes for resource-constrained setups. These values can be adjusted for faster processing. PyTorch Lightning is used for easier integration with Deepspeed, providing a streamlined setup and cleaner code. Fine-tuning a PLM on a specific dataset using PLMFit is a streamlined process that can be executed with a single command.

1. **Downstream heads architectures**

Outputs from different PLMs’ encoder layers are used as representations of protein sequence. The original decoder has been discarded and replaced with the task specific downstream head using these embeddings as input features for training. Transformer-based encoder outputs are 2-d matrices ($V_{local}\epsilon R^{sequence length x embedding dimension})$ where each residue (i.e, token) is described by a 1-d numerical vector ($V_{global}\epsilon R^{embedding dimension}$). Prior to inputting these representations into the downstream architecture, it is necessary to transition from local (i.e. token-wise) to global (i.e. sequence-wise) representations, thereby transforming the entire sequence into a feature vector. To achieve this, multiple reduction approaches can be applied. Within the scope of this study, two reduction techniques were assessed, mean- and sum- pooling. By respectively averaging or adding the elements towards the $sequence length$dimension for each position through the embedding dimension 2-d matrices ( $V_{local}$) are transformed to 1-d vector ($V_{global}$).

Leveraging global representations of protein sequences, deep learning-based architectures are trained to address specific tasks. We evaluated two shallow architectures, logistic regression and a two-layer multilayer perceptron (MLP). Our focus was on highlighting the information encapsulated in the protein language models' (PLMs) embeddings , thus we utilized architectures that do not require extensive optimization as downstream models.

1. **Training and hyperparameter tuning**

Multiple (>3000) setups were assessed in this study. All models were developed using the PyTorch library. For every training procedure Adam optimizer is used with early stopping on best validation loss. All one-hot-encoding baselines and FE-based models have been tuned for optimal hyperparameters (i.e. learning rate, batch size, weight decay) using the Bayesian Optimization algorithm. FT implemented using the Deepspeed (Table S1) package and multiple hyperparameters have been assessed based on trial and error and existing literature. For LoRA, ranks of 4, 8, 16 and batch sizes of 2, 4, 6, 16, 32 were tested before concluding to the final hyperparameters. Similarly, for adapters, bottleneck dimensions of 16, 32, 64 and batch sizes of 4, 8, 16, 32 were examined before concluding to the ones used. All pre-trained PLMs downloaded either from HuggingFace or their original repo and adjusted to allow high-throughput Transfer Learning. All training hyperparameters used for each TL setup are shown in Table S2.


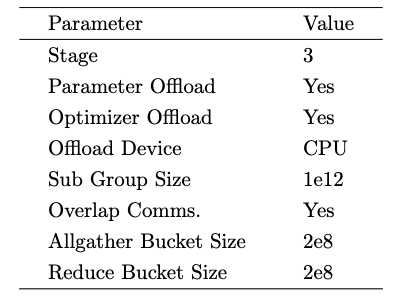


**Table S1. Deepspeed parameters used in PLMFit for fine-tuning setups.**


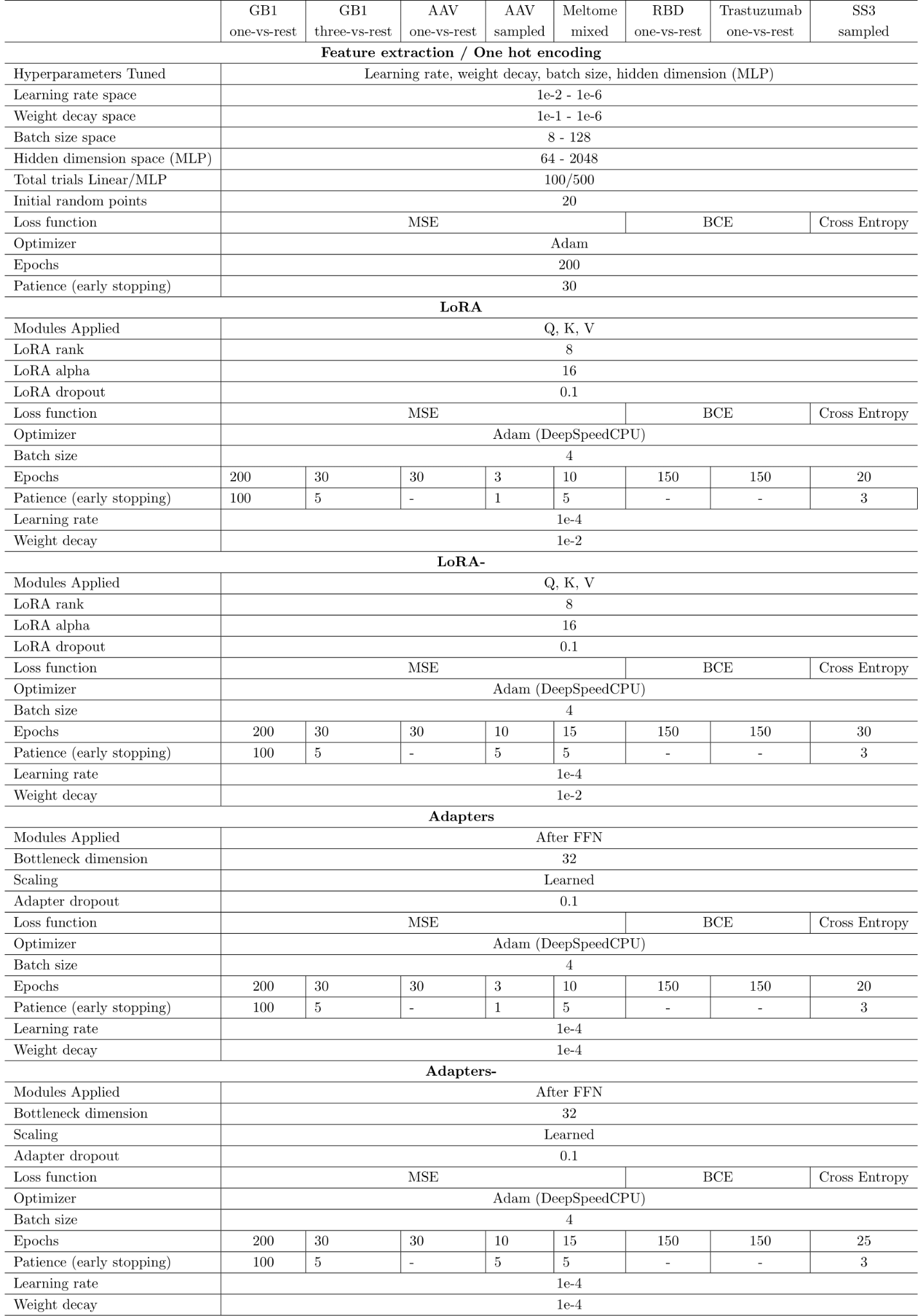


**Table S2. Hyperparameters used for all setups across the different tasks and methods.**

1. **Hardware resources**

For all experiments, we utilized ETH's high-performance computing cluster, Euler. The choice of hardware setup varied based on dataset size and transfer learning techniques. Multiple Nvidia GPUs (GeForce RTX 2080 Ti, RTX 3090, RTX 4090, TITAN RTX, Quadro RTX 6000, Tesla A100) were utilized for inference and backpropagation with 1 to 4 GPUs used in parallel to accelerate training. Tables S3-S10 list the detailed resources used for each setup. Different pooling techniques require the same amount of resources and are therefore combined.


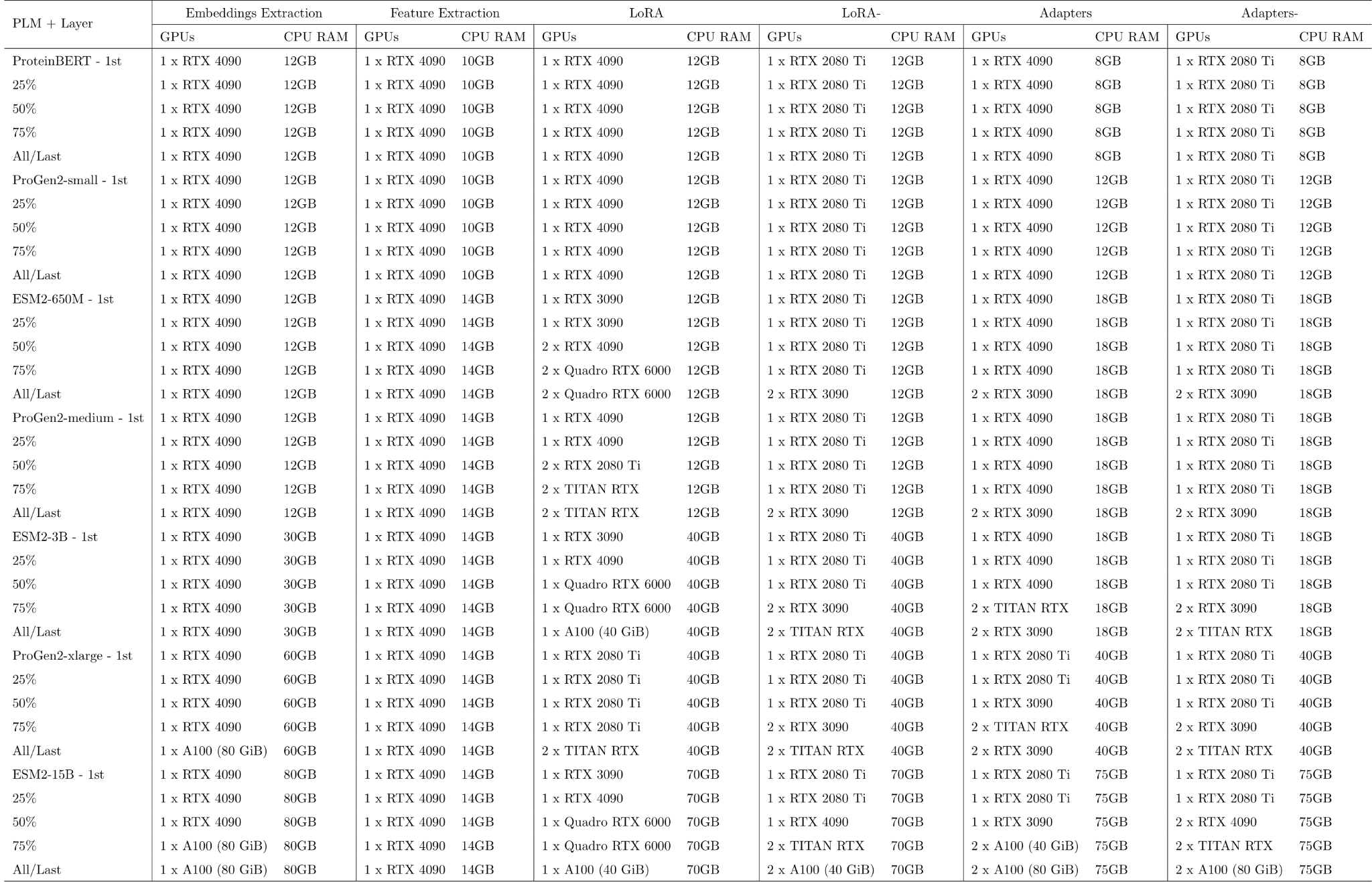


**Table S3. Detailed computational resources (number of GPUs and type, CPU RAM) used for each TL-based model on *GB1-one vs. rest* task.**


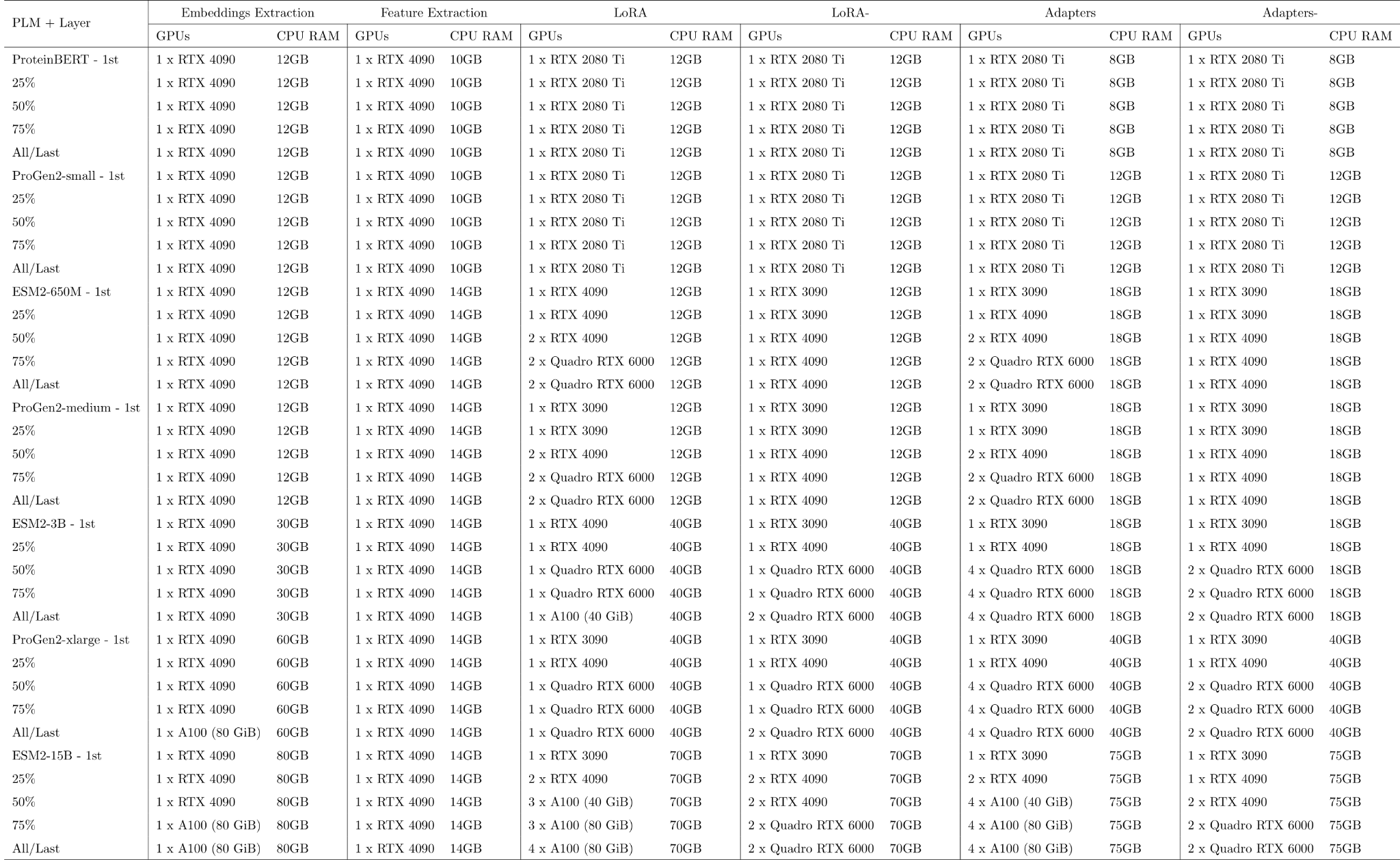


**Table S4. Detailed computational resources (number of GPUs and type, CPU RAM) used for each TL-based model on *GB1-three vs. rest* task.**


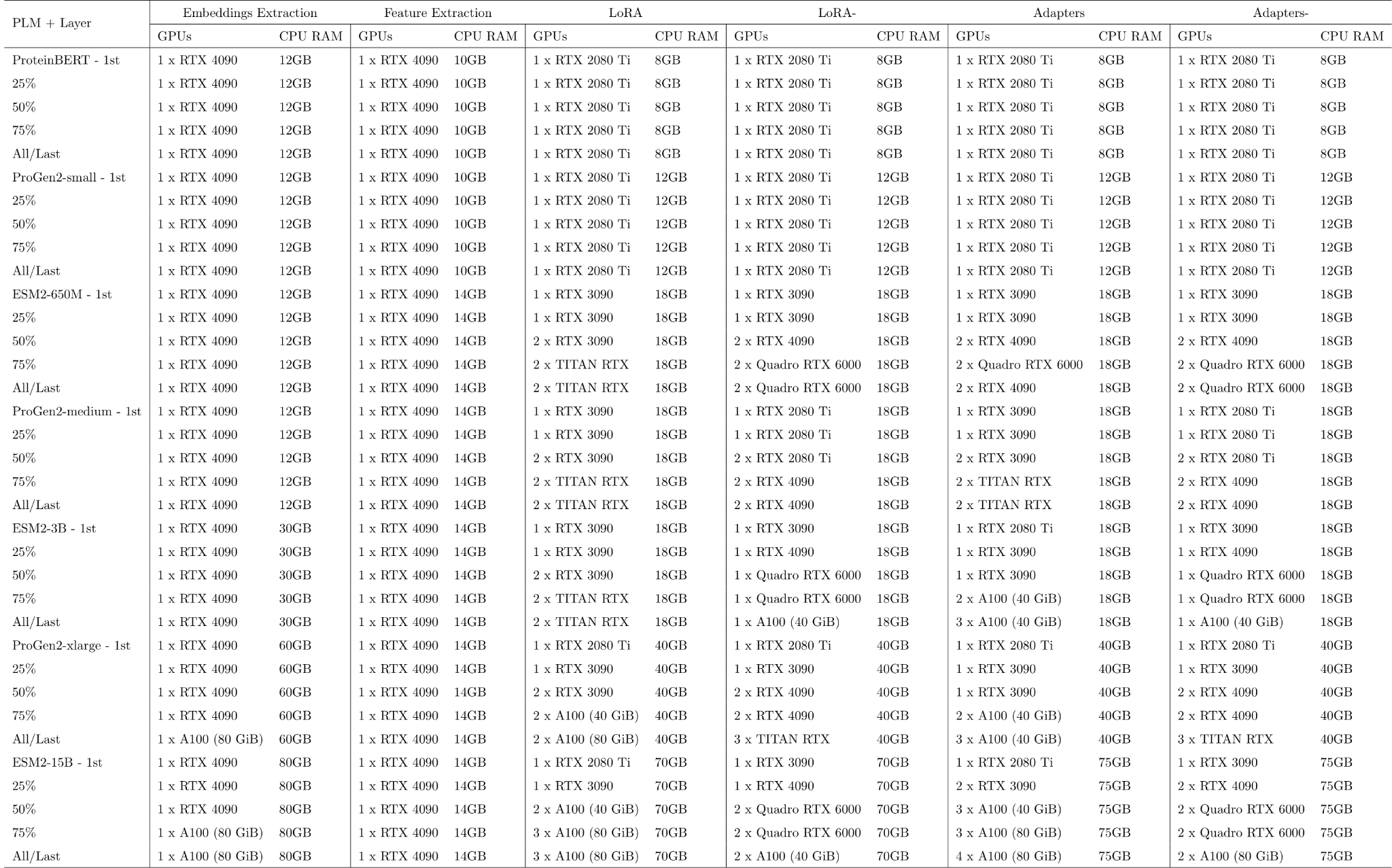


**Table S5. Detailed computational resources (number of GPUs and type, CPU RAM) used for each TL-based model on *AAV-one vs. rest* task.**


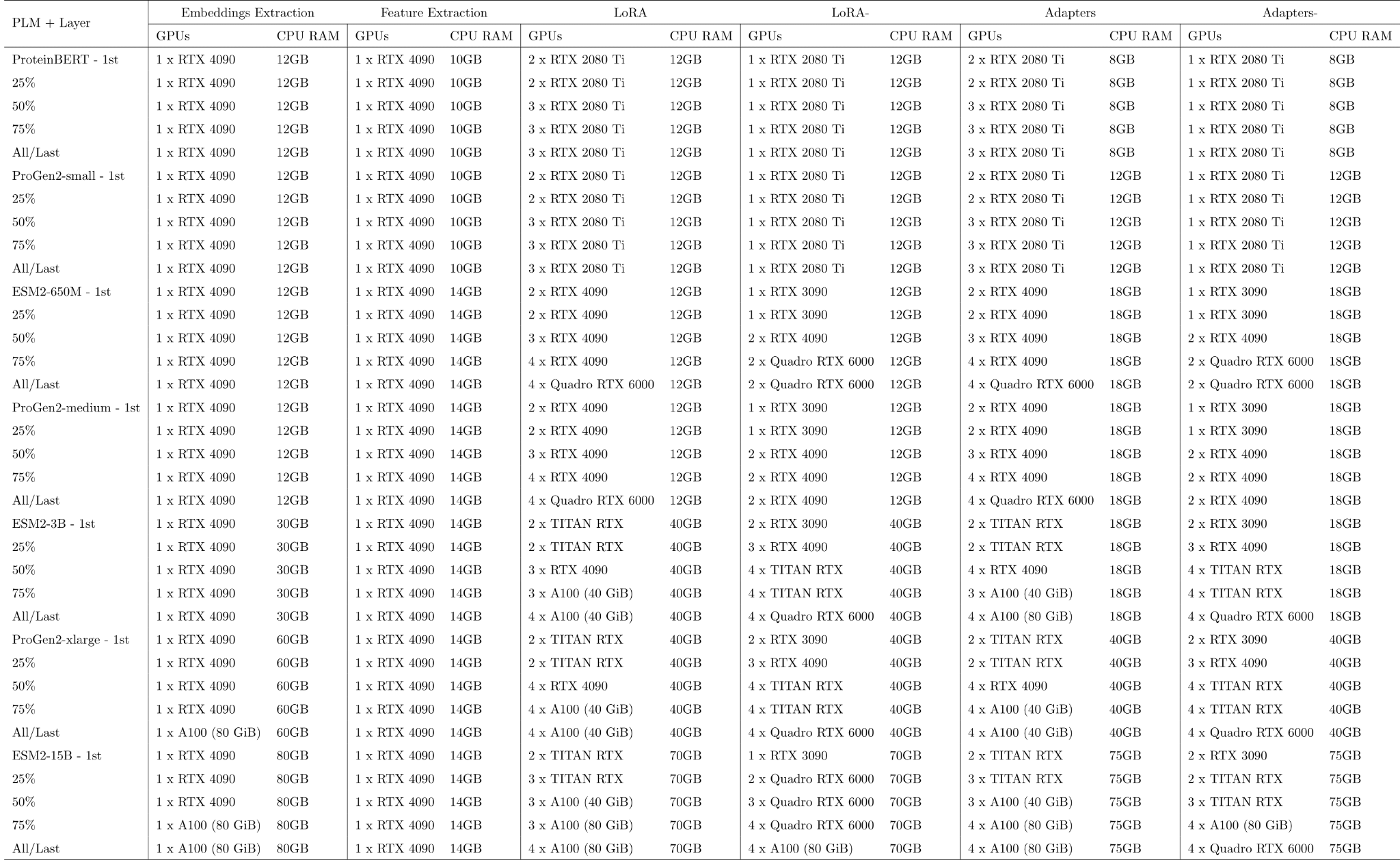


**Table S6. Detailed computational resources (number of GPUs and type, CPU RAM) used for each TL-based model on *AAV-sampled* task.**


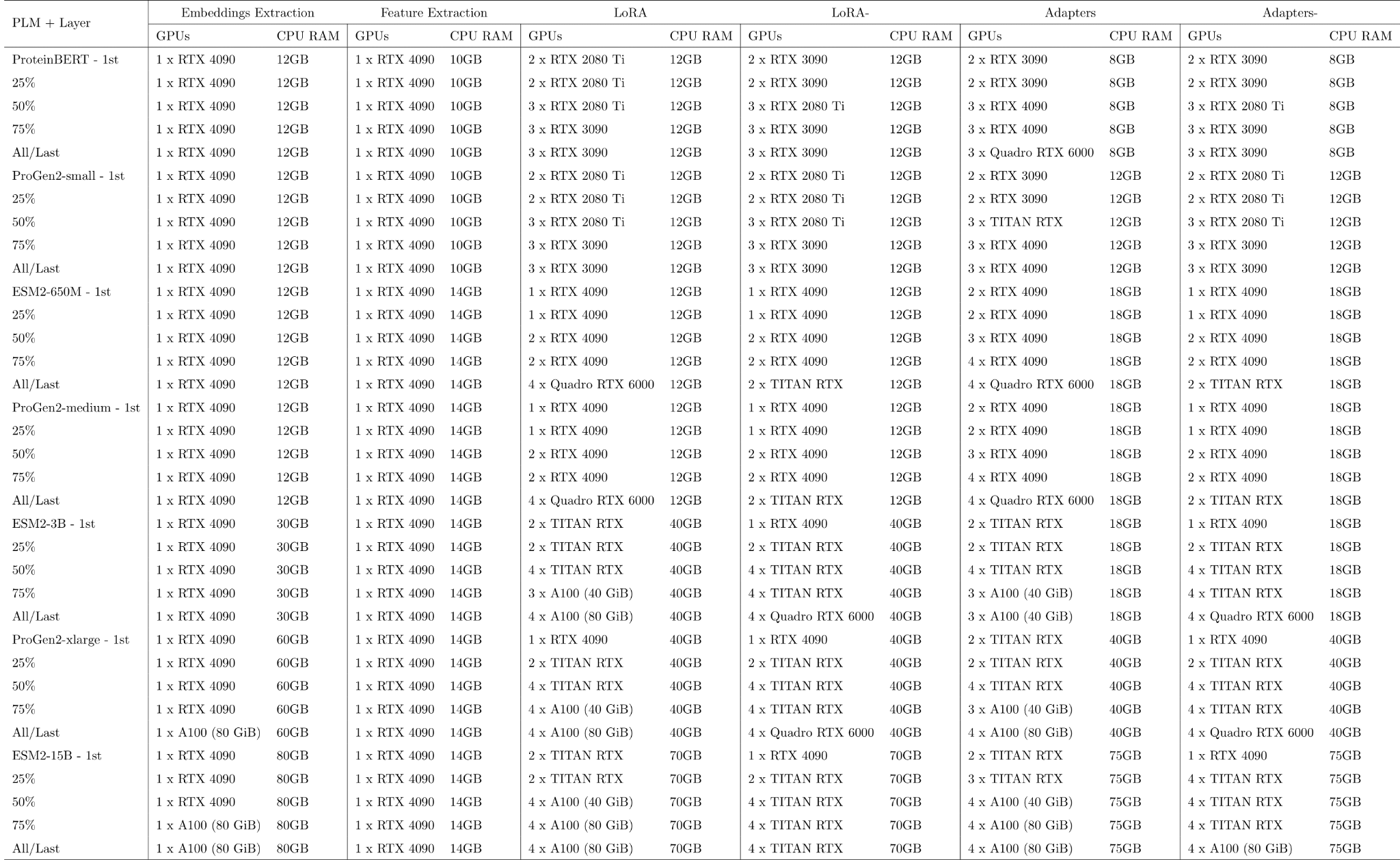


**Table S7.Detailed computational resources (number of GPUs and type, CPU RAM) used for each TL-based model on *Meltome-mixed* task.**


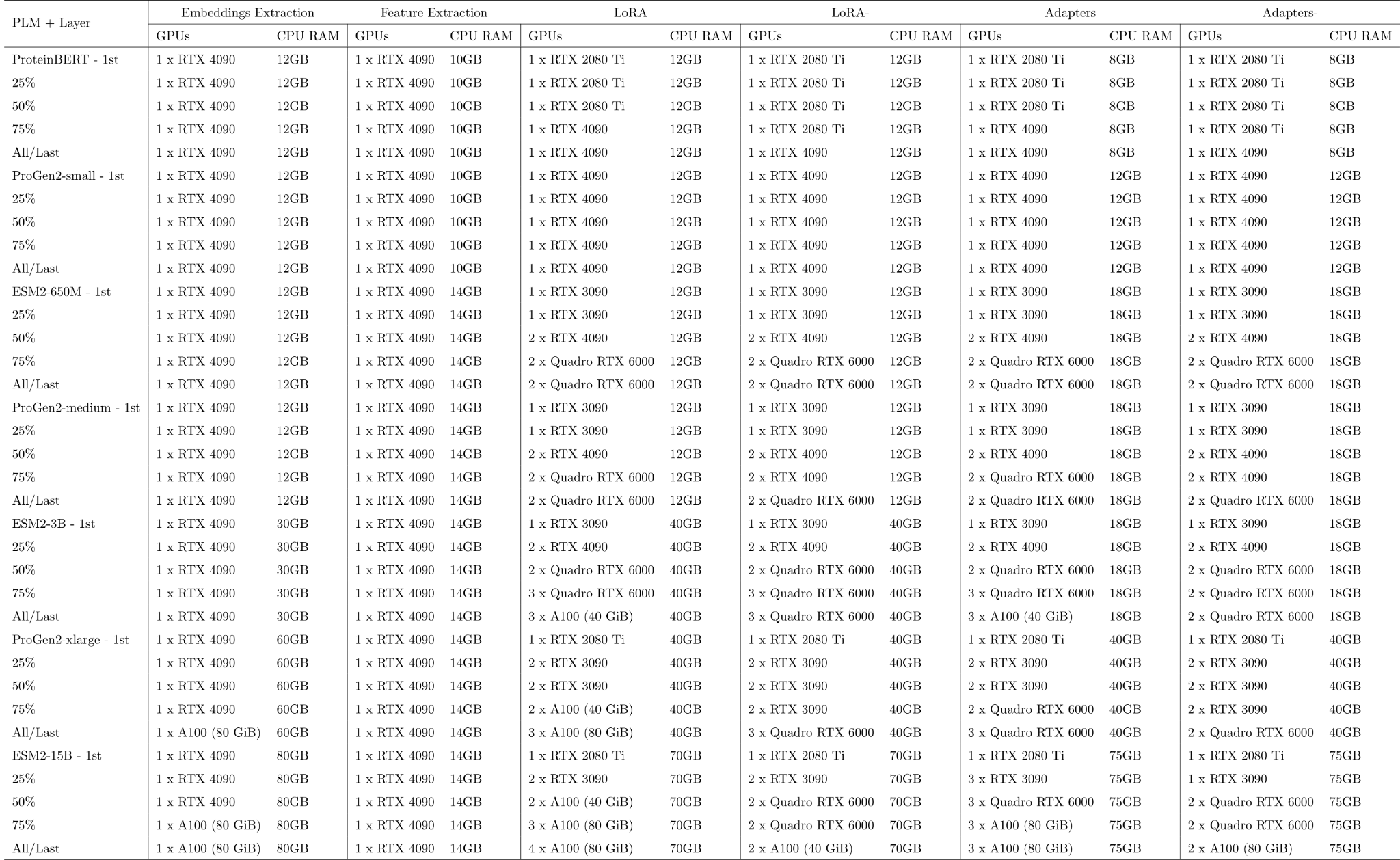


**Table S8. Detailed computational resources (number of GPUs and type, CPU RAM) used for each TL-based model on *RBD-one vs. rest* task.**


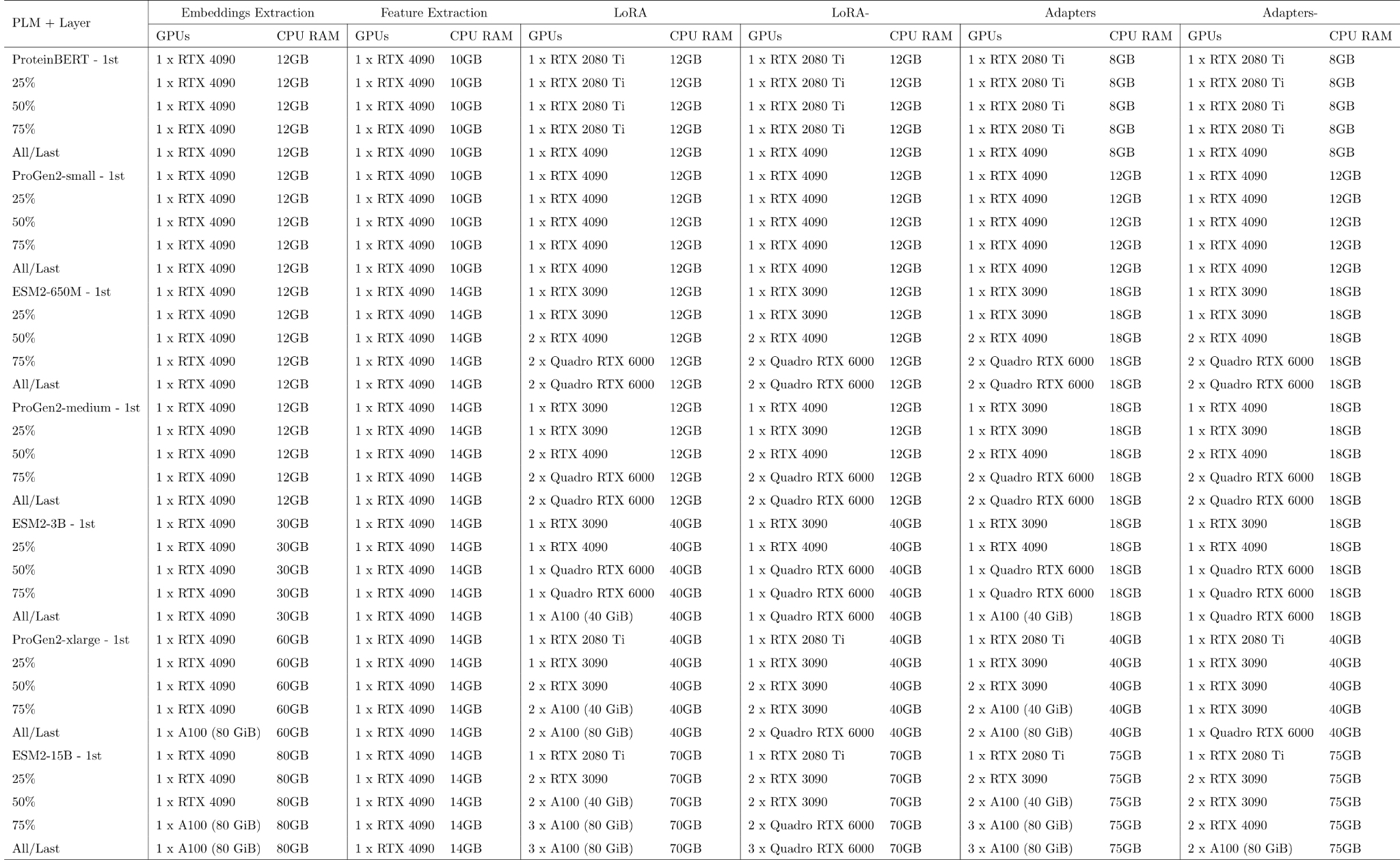


**Table S9. Detailed computational resources (number of GPUs and type, CPU RAM) used for each TL-based model on *Trastuzumab-one vs. rest* task.**


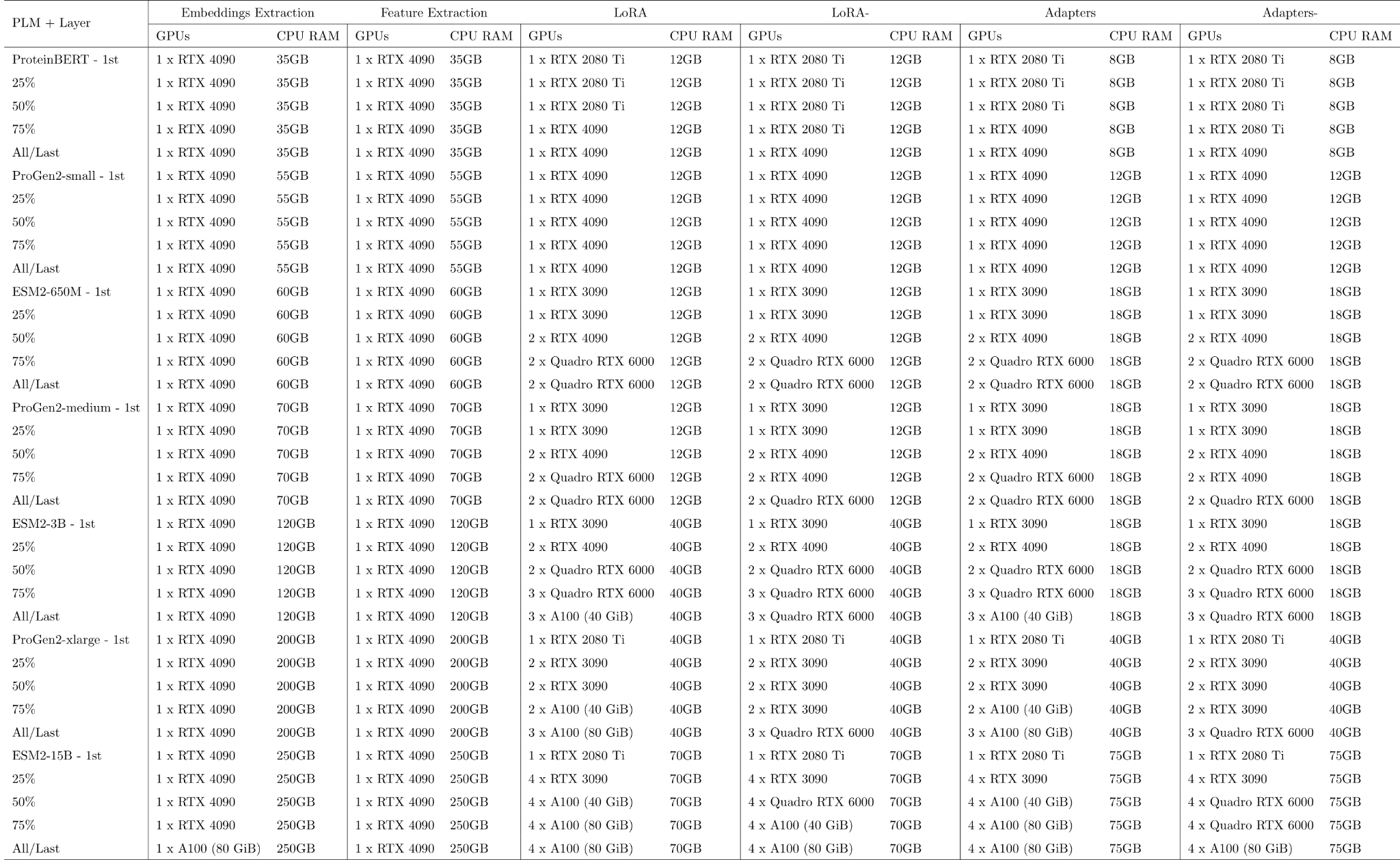


**Table S10. Detailed computational resources (number of GPUs and type, CPU RAM) used for each TL-based model on *SS3-sampled* task.**

1. **Extended results tables**

We present an extended representation of the results, including Table S11 which provides statistical summaries of the box plots in Figure 4B and heatmaps in Figures S2-S9, displaying the result metrics for all 3,150 TL setups evaluated.

**
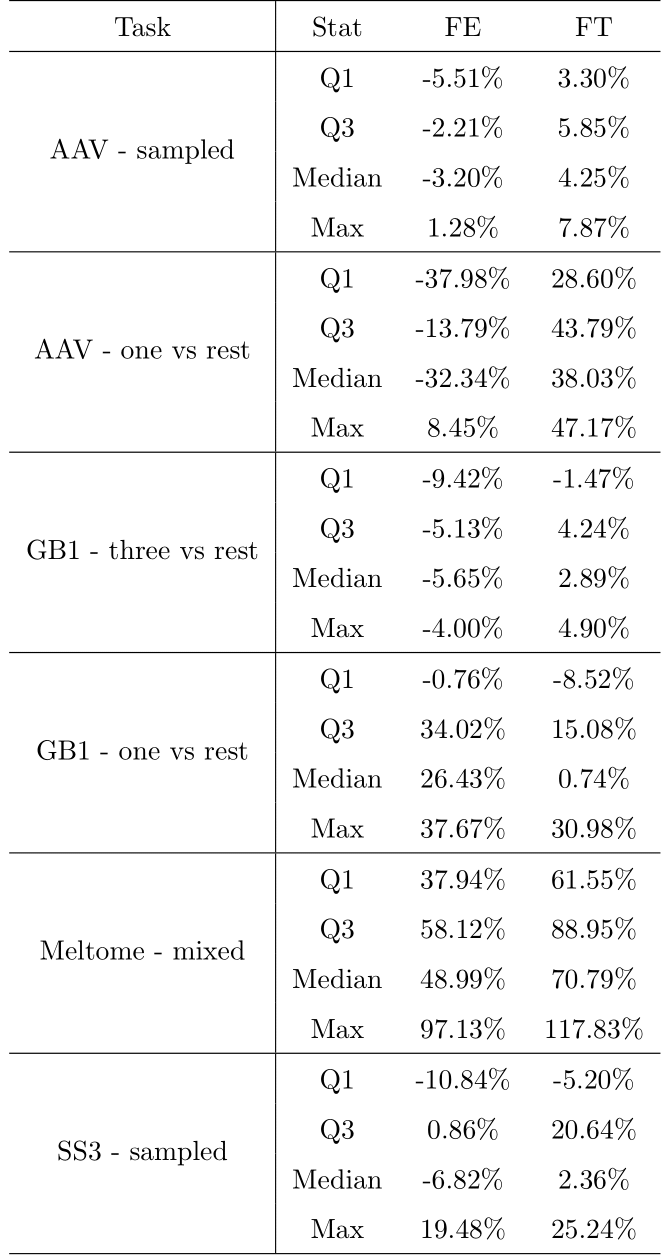
**

**Table S11. Boxplot numerical values of Figure 4B.**


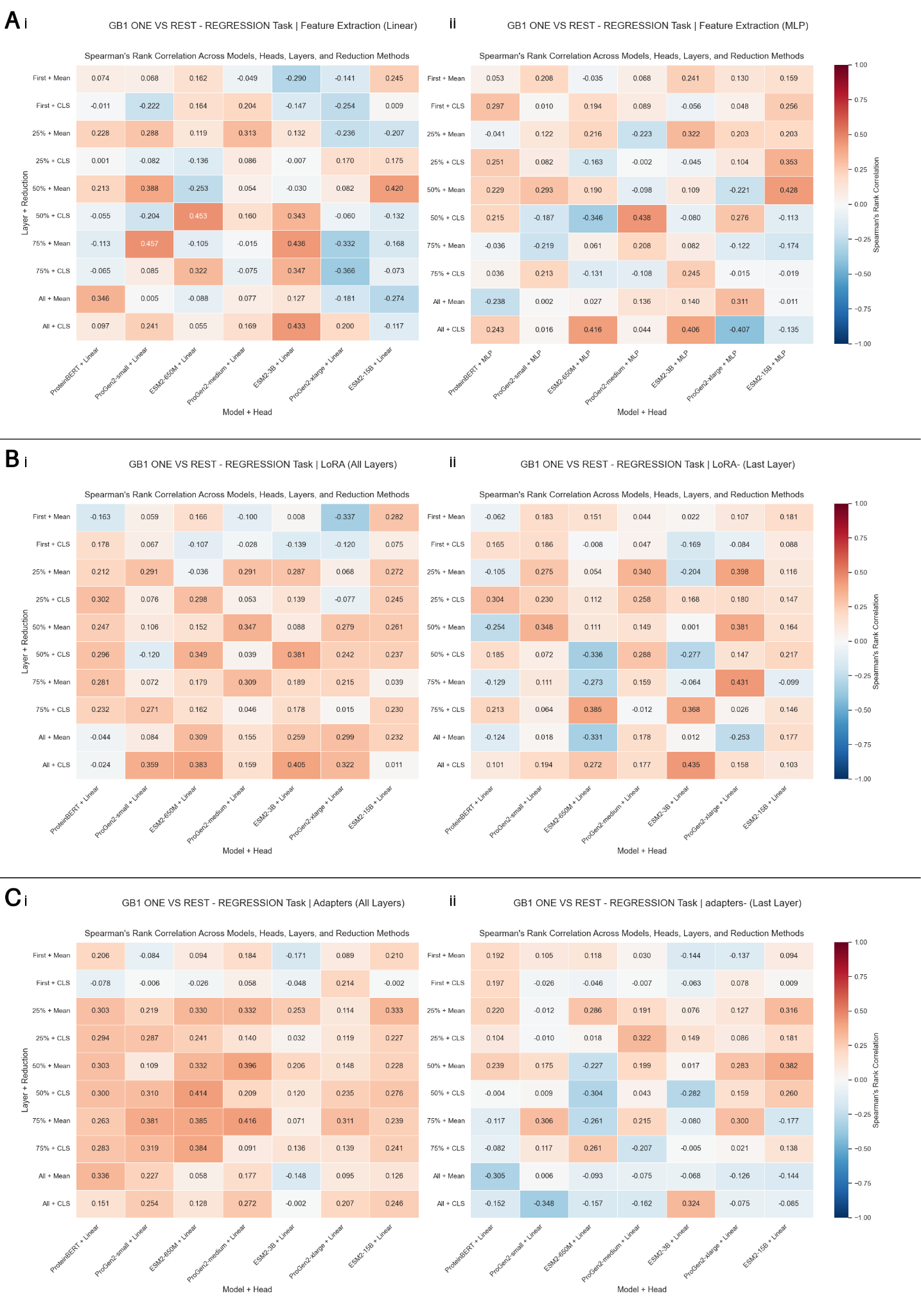


**Figure S2. Detailed results for *GB1-one vs. rest* task.** Spearman’s rank correlation is used as a performance metric for each setup, with x-axis showing the PLM and the head used and the y-axis representing the layers used and the pooling method employed; mean stands for mean pooling and CLS for pooling the classification token for BERT-based PLMs (ESM2, ProteinBERT) and the EOS token for GPT-based PLMs (ProGen2). (A) Feature extraction detailed results using (i) a linear downstream head and (ii) a MLP with one hidden layer as a downstream head. (B) LoRA detailed results when (i) applying LoRA to all layers of PLMs and (ii) applying LoRA to the last layer of PLMs. (C) Adapters detailed results when (i) applying adapters to all layers of PLMs and (ii) applying adapters to the last layer of PLMs. Empty cells represent work in progress, due to the computational burden these setups bear.

TL: Transfer Learning; PLM: Protein Language Model; LoRA: Low Rank Adaptation


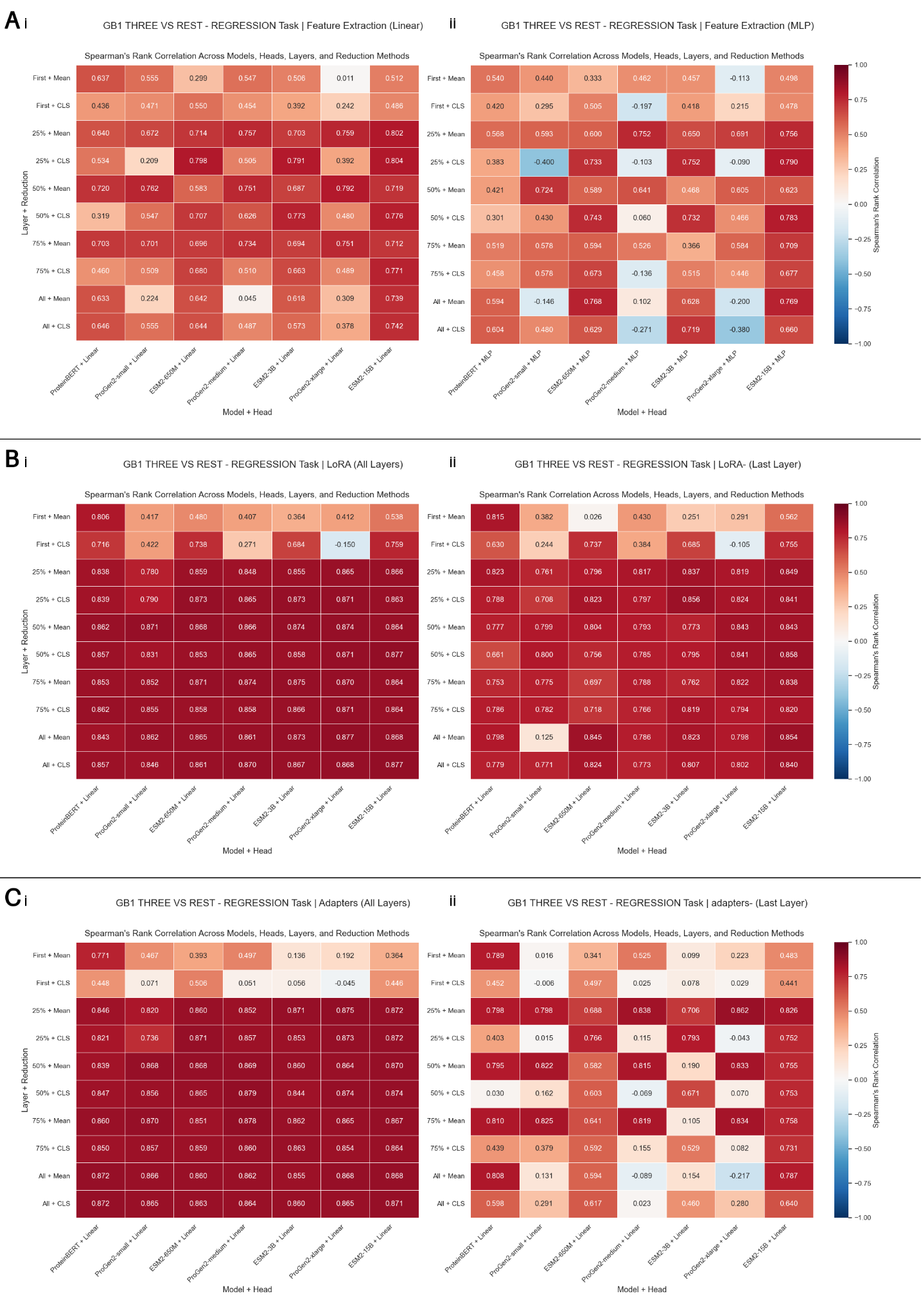


**Figure S3. Detailed results for *GB1-three vs. rest* task.** Spearman’s rank correlation is used as a performance metric for each setup, with x-axis showing the PLM and the head used and the y-axis representing the layers used and the pooling method employed; mean stands for mean pooling and CLS for pooling the classification token for BERT-based PLMs (ESM2, ProteinBERT) and the EOS token for GPT-based PLMs (ProGen2). (A) Feature extraction detailed results using (i) a linear downstream head and (ii) a MLP with one hidden layer as a downstream head. (B) LoRA detailed results when (i) applying LoRA to all layers of PLMs and (ii) applying LoRA to the last layer of PLMs. (C) Adapters detailed results when (i) applying adapters to all layers of PLMs and (ii) applying adapters to the last layer of PLMs. Empty cells represent work in progress, due to the computational burden these setups bear.

TL: Transfer Learning; PLM: Protein Language Model; LoRA: Low Rank Adaptation


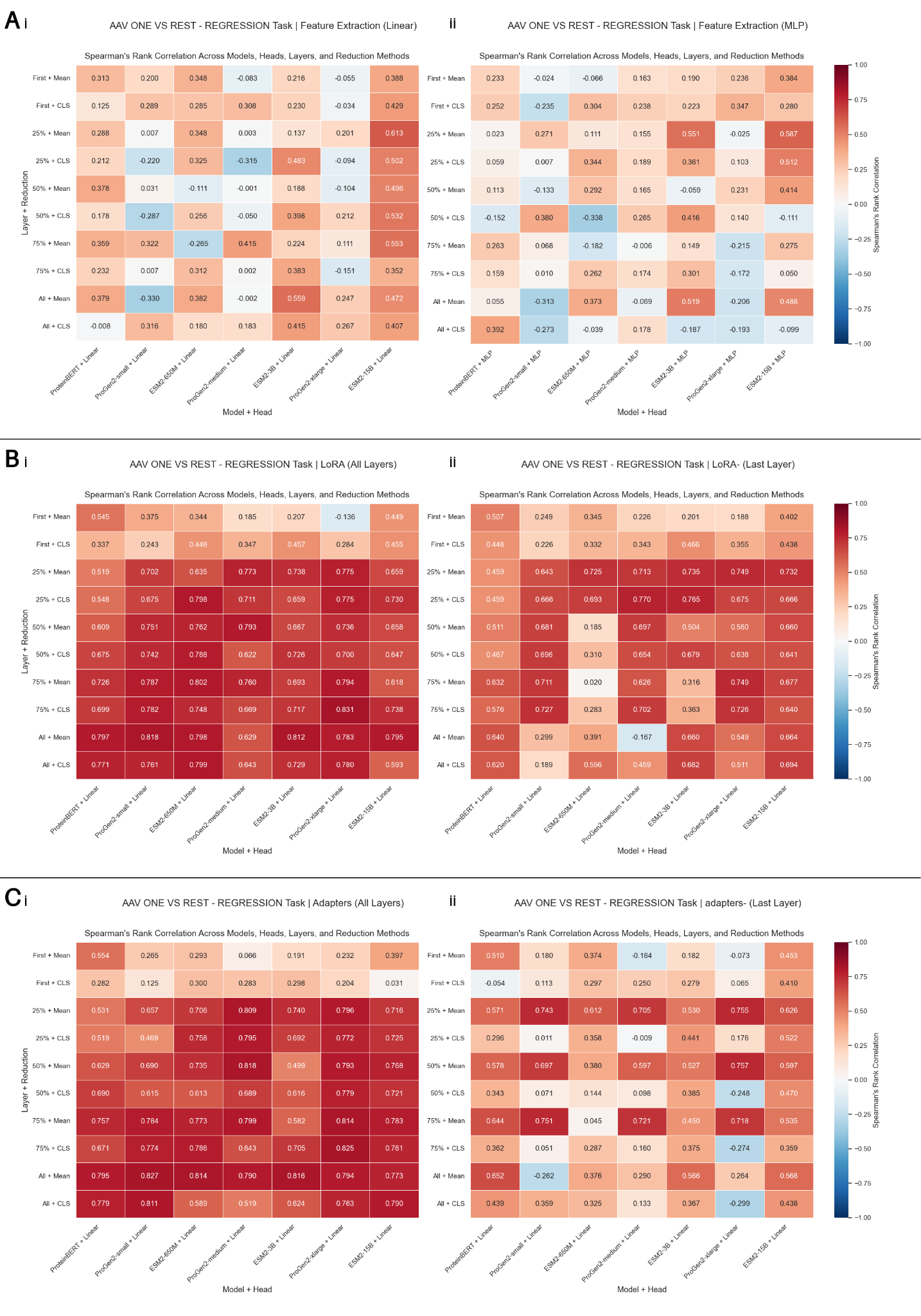


**Figure S4. Detailed results for *AAV-one vs. rest* task.**

Spearman’s rank correlation is used as a performance metric for each setup, with x-axis showing the PLM and the head used and the y-axis representing the layers used and the pooling method employed; mean stands for mean pooling and CLS for pooling the classification token for BERT-based PLMs (ESM2, ProteinBERT) and the EOS token for GPT-based PLMs (ProGen2). (A) Feature extraction detailed results using (i) a linear downstream head and (ii) a MLP with one hidden layer as a downstream head. (B) LoRA detailed results when (i) applying LoRA to all layers of PLMs and (ii) applying LoRA to the last layer of PLMs. (C) Adapters detailed results when (i) applying adapters to all layers of PLMs and (ii) applying adapters to the last layer of PLMs. Empty cells represent work in progress, due to the computational burden these setups bear.

TL: Transfer Learning; PLM: Protein Language Model; LoRA: Low Rank Adaptation


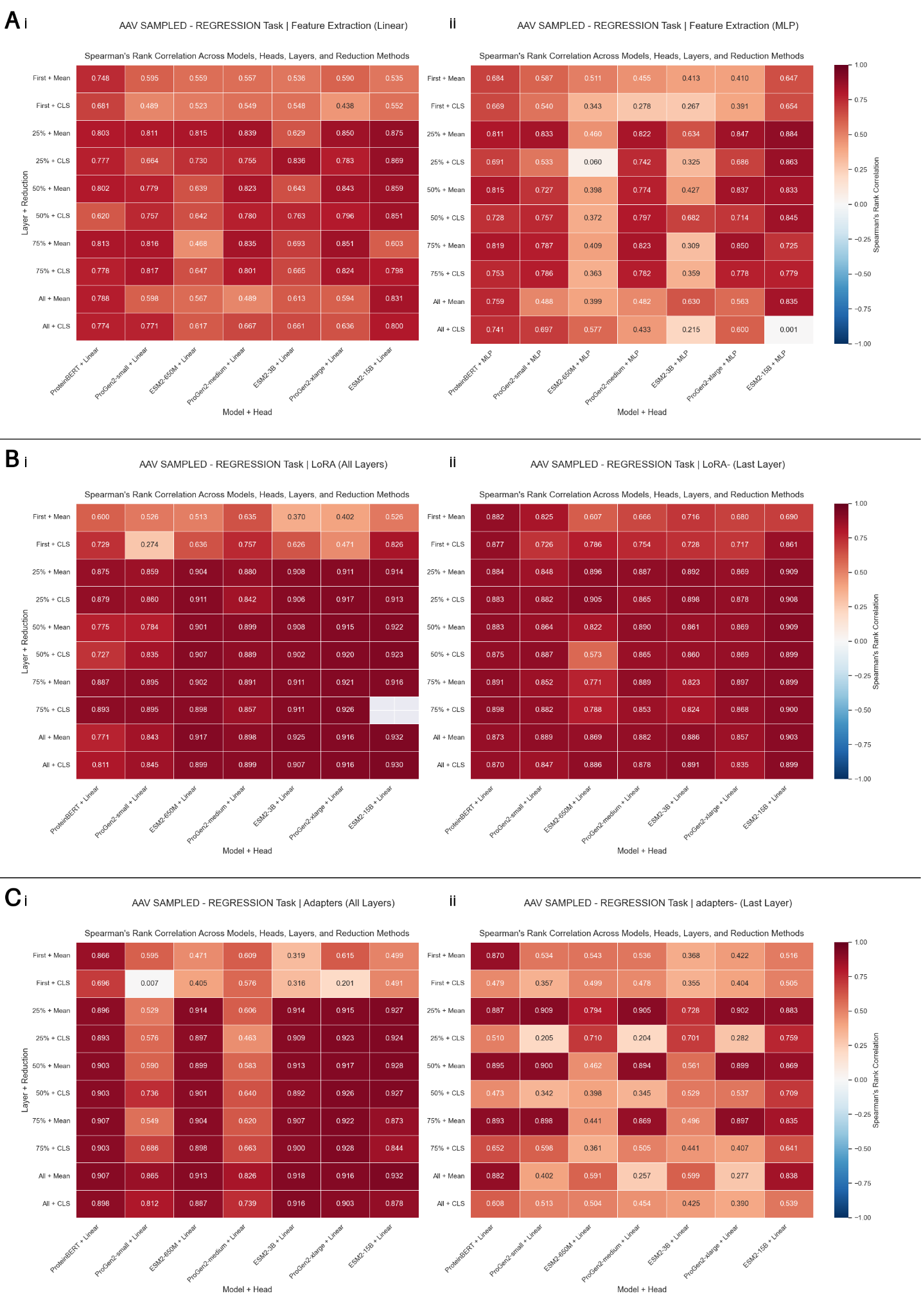


**Figure S5. Detailed results for *AAV-sampled* task.** Spearman’s rank correlation is used as a performance metric for each setup, with x-axis showing the PLM and the head used and the y-axis representing the layers used and the pooling method employed; mean stands for mean pooling and CLS for pooling the classification token for BERT-based PLMs (ESM2, ProteinBERT) and the EOS token for GPT-based PLMs (ProGen2). (A) Feature extraction detailed results using (i) a linear downstream head and (ii) a MLP with one hidden layer as a downstream head. (B) LoRA detailed results when (i) applying LoRA to all layers of PLMs and (ii) applying LoRA to the last layer of PLMs. (C) Adapters detailed results when (i) applying adapters to all layers of PLMs and (ii) applying adapters to the last layer of PLMs. Empty cells represent work in progress, due to the computational burden these setups bear.

TL: Transfer Learning; PLM: Protein Language Model; LoRA: Low Rank Adaptation


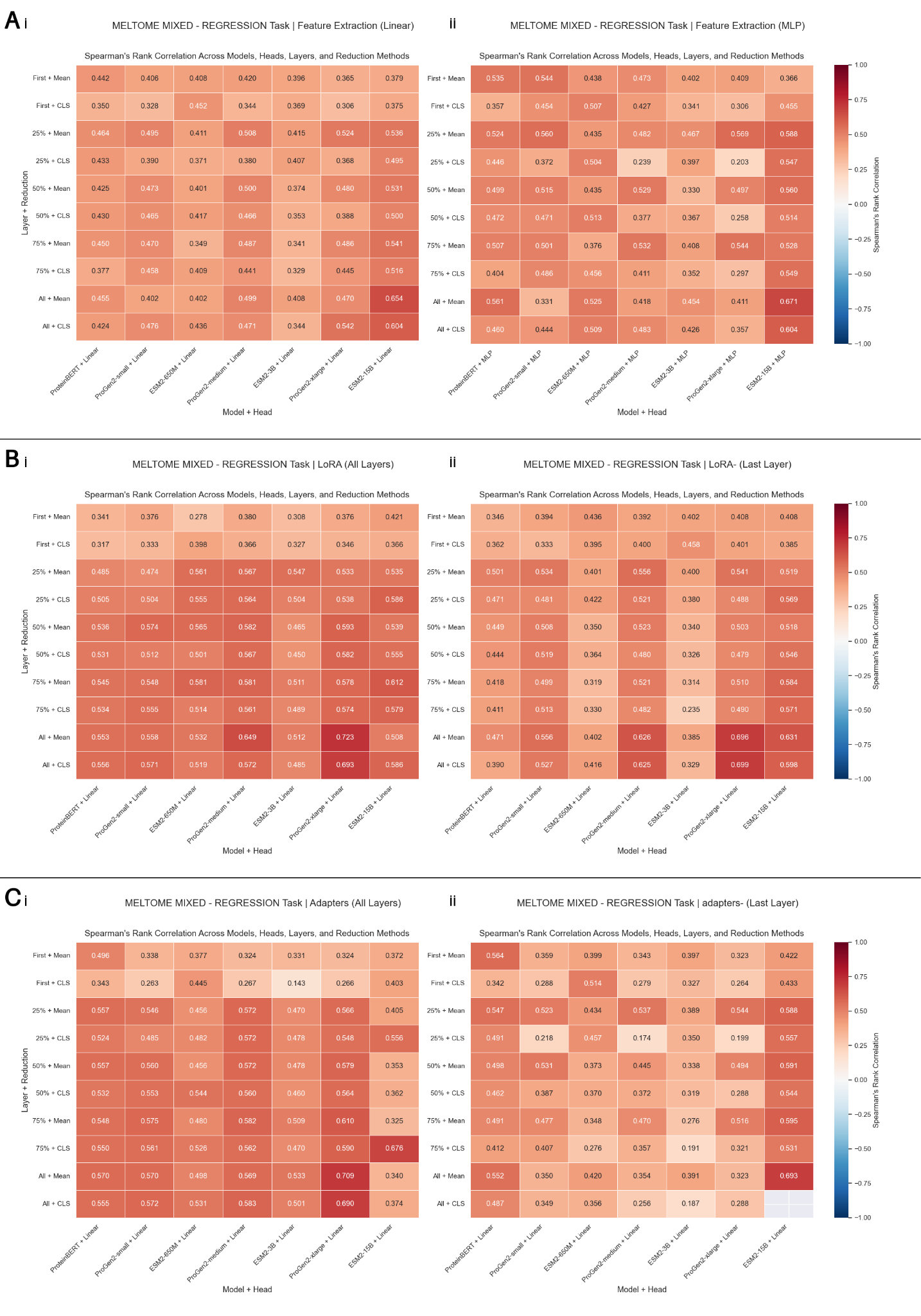


**Figure S6. Detailed results for *Meltome-mixed* task.**

Spearman’s rank correlation is used as a performance metric for each setup, with x-axis showing the PLM and the head used and the y-axis representing the layers used and the pooling method employed; mean stands for mean pooling and CLS for pooling the classification token for BERT-based PLMs (ESM2, ProteinBERT) and the EOS token for GPT-based PLMs (ProGen2). (A) Feature extraction detailed results using (i) a linear downstream head and (ii) a MLP with one hidden layer as a downstream head. (B) LoRA detailed results when (i) applying LoRA to all layers of PLMs and (ii) applying LoRA to the last layer of PLMs. (C) Adapters detailed results when (i) applying adapters to all layers of PLMs and (ii) applying adapters to the last layer of PLMs. Empty cells represent work in progress, due to the computational burden these setups bear.

TL: Transfer Learning; PLM: Protein Language Model; LoRA: Low Rank Adaptation


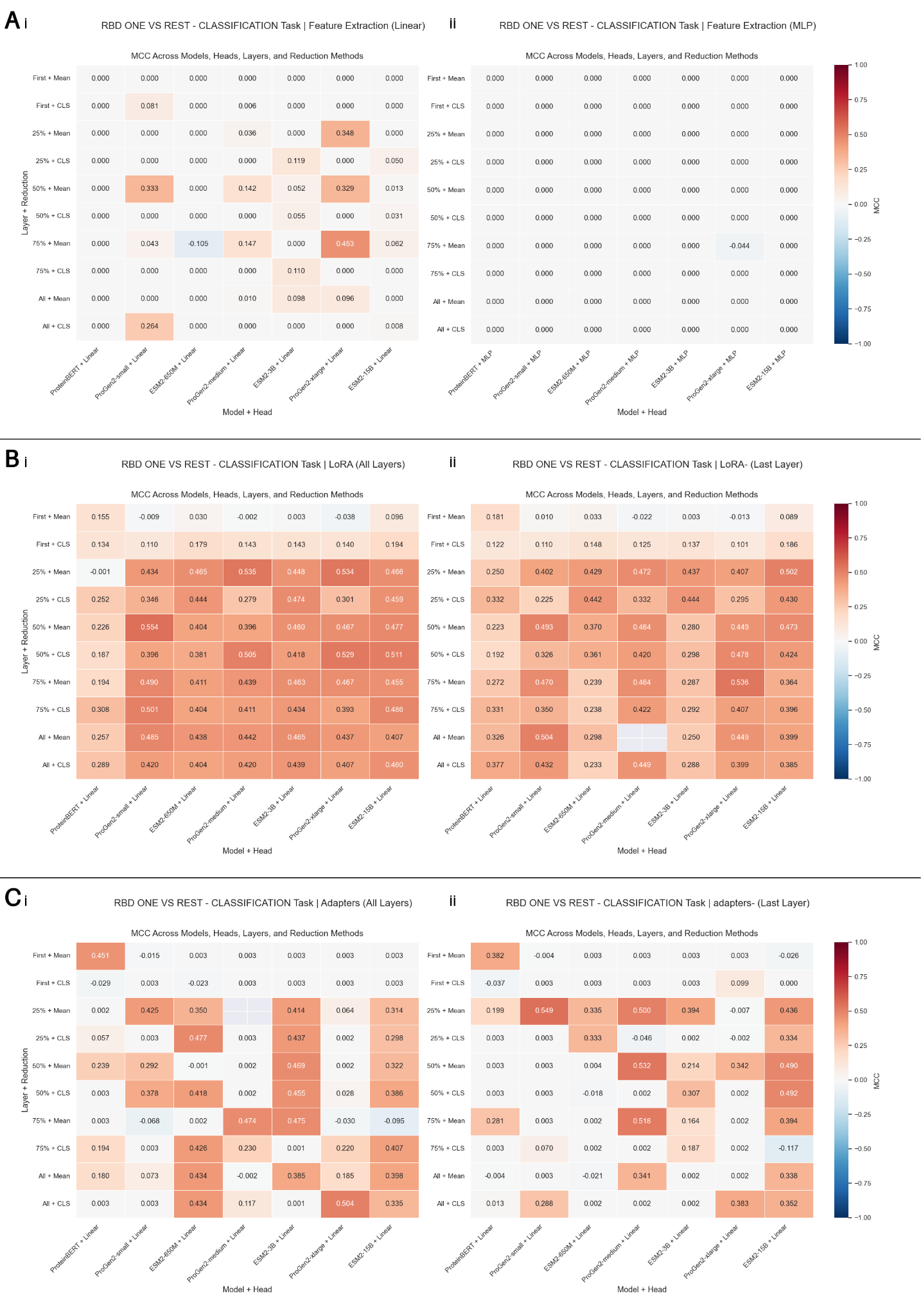


**Figure S7. Detailed results for *RBD-three vs. rest* task.**

MCC is used as a performance metric for each setup, with x-axis showing the PLM and the head used and the y-axis representing the layers used and the pooling method employed; mean stands for mean pooling and CLS for pooling the classification token for BERT-based PLMs (ESM2, ProteinBERT) and the EOS token for GPT-based PLMs (ProGen2). (A) Feature extraction detailed results using (i) a linear downstream head and (ii) a MLP with one hidden layer as a downstream head. (B) LoRA detailed results when (i) applying LoRA to all layers of PLMs and (ii) applying LoRA to the last layer of PLMs. (C) Adapters detailed results when (i) applying adapters to all layers of PLMs and (ii) applying adapters to the last layer of PLMs. Empty cells represent work in progress, due to the computational burden these setups bear.

TL: Transfer Learning; PLM: Protein Language Model; LoRA: Low Rank Adaptation


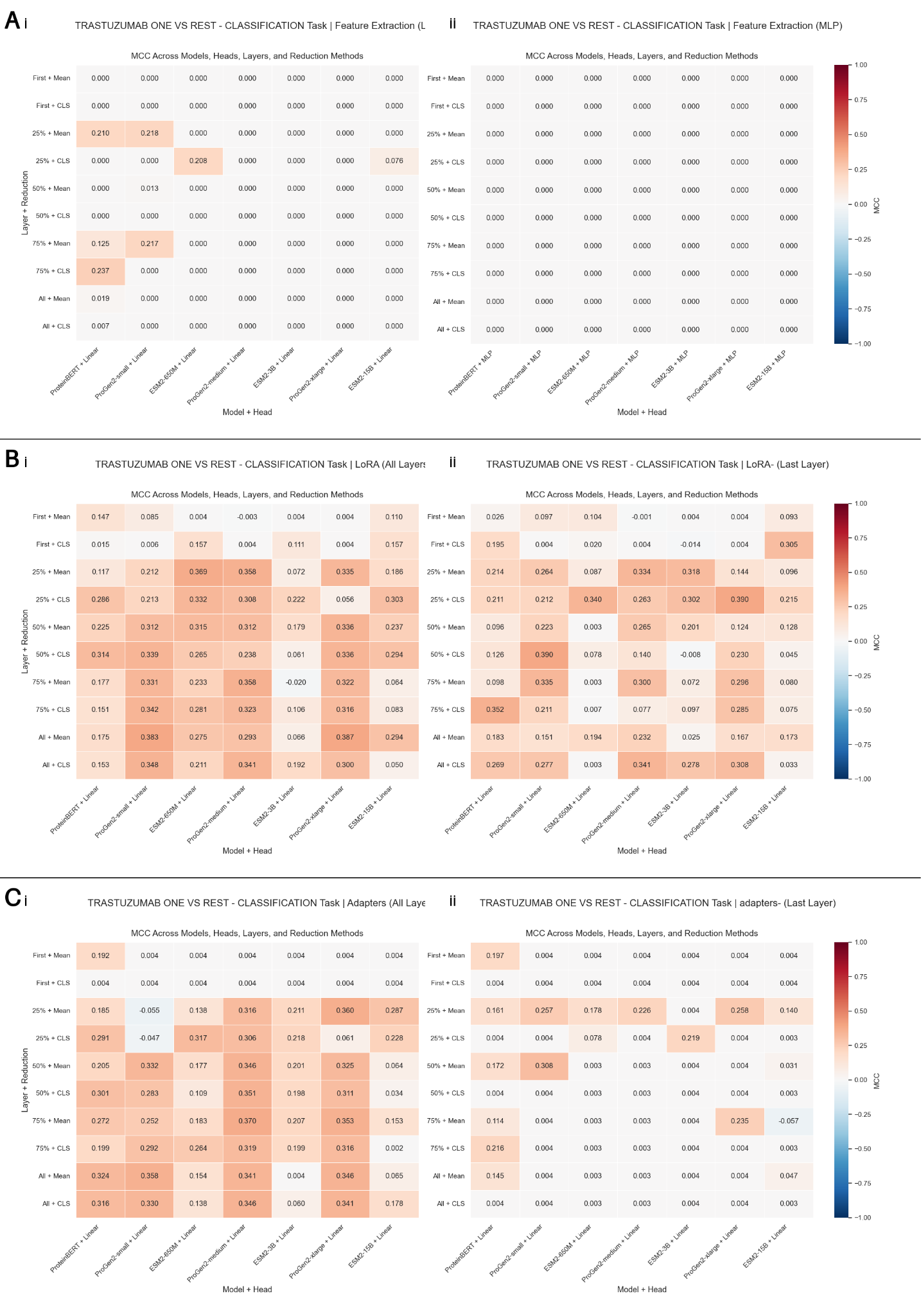


**Figure S8. Detailed results for *Trastuzumab-three vs. rest* task.**

MCC is used as a performance metric for each setup, with x-axis showing the PLM and the head used and the y-axis representing the layers used and the pooling method employed; mean stands for mean pooling and CLS for pooling the classification token for BERT-based PLMs (ESM2, ProteinBERT) and the EOS token for GPT-based PLMs (ProGen2). (A) Feature extraction detailed results using (i) a linear downstream head and (ii) a MLP with one hidden layer as a downstream head. (B) LoRA detailed results when (i) applying LoRA to all layers of PLMs and (ii) applying LoRA to the last layer of PLMs. (C) Adapters detailed results when (i) applying adapters to all layers of PLMs and (ii) applying adapters to the last layer of PLMs. Empty cells represent work in progress, due to the computational burden these setups bear.

TL: Transfer Learning; PLM: Protein Language Model; LoRA: Low Rank Adaptation


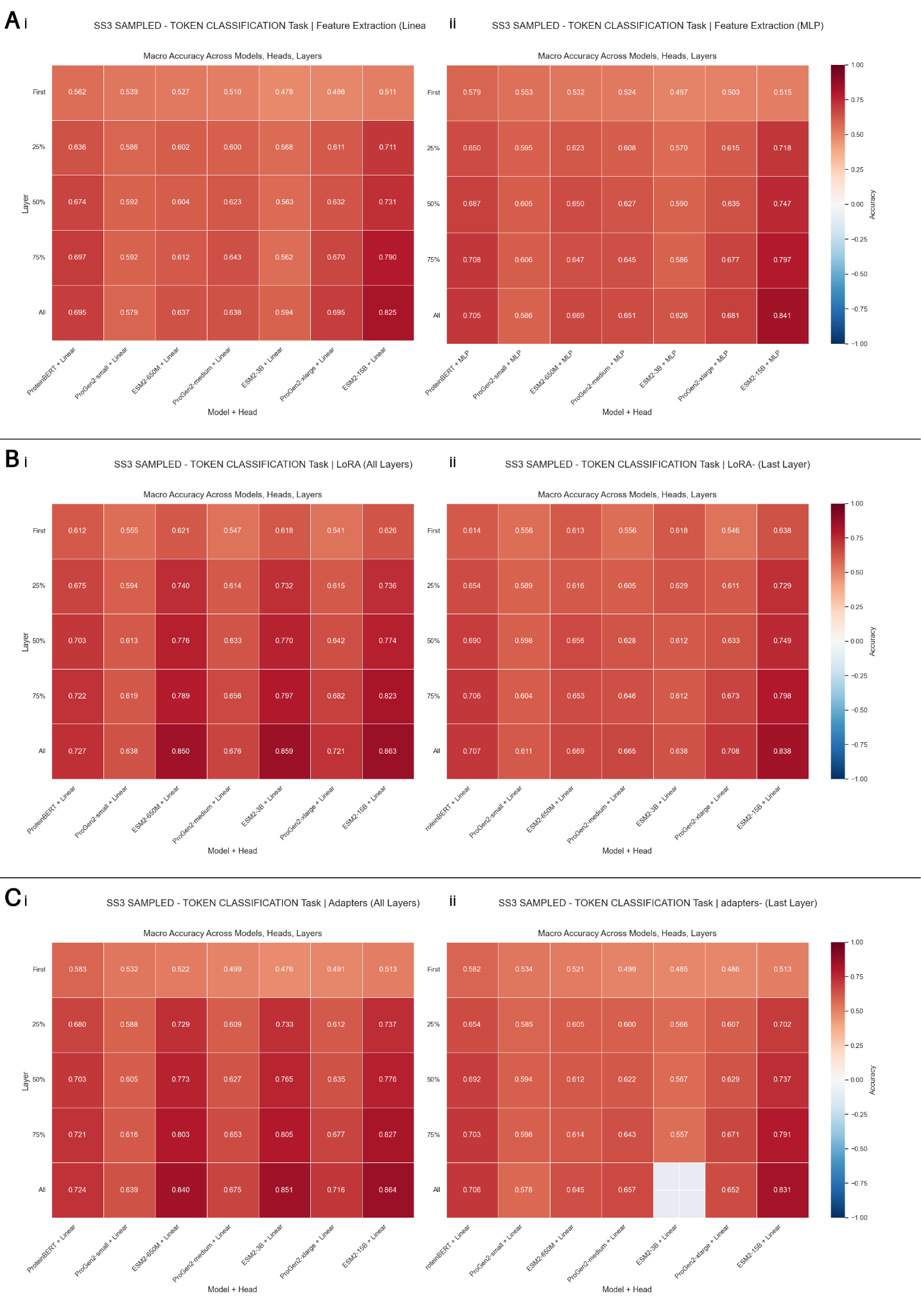


**Figure S9. Detailed results for *SS3-sampled* task.**

Spearman’s rank correlation is used as a performance metric for each setup, with x-axis showing the PLM and the head used and the y-axis representing the layers used; no pooling was performed. (A) Feature extraction detailed results using (i) a linear downstream head and (ii) a MLP with one hidden layer as a downstream head. (B) LoRA detailed results when (i) applying LoRA to all layers of PLMs and (ii) applying LoRA to the last layer of PLMs. (C) Adapters detailed results when (i) applying adapters to all layers of PLMs and (ii) applying adapters to the last layer of PLMs. Empty cells represent work in progress, due to the computational burden these setups bear.

TL: Transfer Learning; PLM: Protein Language Model; LoRA: Low Rank Adaptation
